# Warming and redistribution of nitrogen inputs drive an increase in terrestrial nitrous oxide emission factor

**DOI:** 10.1038/s41467-022-32001-z

**Published:** 2022-07-25

**Authors:** E. Harris, L. Yu, Y-P. Wang, J. Mohn, S. Henne, E. Bai, M. Barthel, M. Bauters, P. Boeckx, C. Dorich, M. Farrell, P. B. Krummel, Z. M. Loh, M. Reichstein, J. Six, M. Steinbacher, N. S. Wells, M. Bahn, P. Rayner

**Affiliations:** 1 Swiss Data Science Centre, ETH Zurich, 8092 Zurich, Switzerland; 2 Functional Ecology Research Group, Institute of Ecology, University of Innsbruck, 6020 Innsbruck, Austria; 3 Institute of Environment and Ecology, Tsinghua Shenzhen International Graduate School (SIGS), Tsinghua University, Shenzhen, 518055 China; 4 Laboratory for Air Pollution & Environmental Technology, Empa, Swiss Federal Laboratories for Materials Science and Technology, 8600 Duebendorf, Switzerland; 5 Climate Science Centre, CSIRO Oceans and Atmosphere, Aspendale, VIC 3195 Australia; 6 Key Laboratory of Geographical Processes and Ecological Security of Changbai Mountains, Ministry of Education, School of Geographical Sciences, Northeast Normal University, Changchun, 130024 China; 7 Department of Environmental Systems Science, ETH Zurich, 8092 Zurich, Switzerland; 8 Isotope Bioscience Laboratory - ISOFYS, Department of Green Chemistry and Technology, Ghent University, Coupure Links 653, 9000 Ghent, Belgium; 9 Natural Resource Ecology Laboratory, Colorado State University, Fort Collins, 80523 CO USA; 10 CSIRO Agriculture and Food, Locked bag 2, Glen Osmond, SA 5064 Australia; 11 Department of Biogeochemical Integration, Max Planck Institute for Biogeochemistry, Jena, Germany; 12 Centre for Coastal Biogeochemistry, Southern Cross University, Lismore, NSW 2480 Australia; 13 Department of Soil and Physical Sciences, Agriculture and Life Sciences, Lincoln University, Lincoln, 7647 New Zealand; 14 School of Geography, Earth and Atmospheric Sciences, University of Melbourne, Parkville, VIC 3052 Australia; 15 Melbourne Climate Futures Climate and Energy College, University of Melbourne, Parkville, VIC 3052 Australia

**Keywords:** Element cycles, Element cycles, Agriculture, Ecological modelling, Stable isotope analysis

## Abstract

Anthropogenic nitrogen inputs cause major negative environmental impacts, including emissions of the important greenhouse gas N_2_O. Despite their importance, shifts in terrestrial N loss pathways driven by global change are highly uncertain. Here we present a coupled soil-atmosphere isotope model (IsoTONE) to quantify terrestrial N losses and N_2_O emission factors from 1850-2020. We find that N inputs from atmospheric deposition caused 51% of anthropogenic N_2_O emissions from soils in 2020. The mean effective global emission factor for N_2_O was 4.3 ± 0.3% in 2020 (weighted by N inputs), much higher than the surface area-weighted mean (1.1 ± 0.1%). Climate change and spatial redistribution of fertilisation N inputs have driven an increase in global emission factor over the past century, which accounts for 18% of the anthropogenic soil flux in 2020. Predicted increases in fertilisation in emerging economies will accelerate N_2_O-driven climate warming in coming decades, unless targeted mitigation measures are introduced.

## Abstract (web summary)

Soil nitrogen isotopic composition is used to drive the IsoTONE model, which is constrained with measurements of tropospheric nitrous oxide isotopic composition. The model results reveal causes of rising mean global nitrous oxide emission factor.

## Introduction

Nitrous oxide (N_2_O) is a long-lived greenhouse gas and a key stratospheric ozone-depleting substance^1,2^. The atmospheric N_2_O mole fraction has increased from ~270 nmol mol^−1^ in the preindustrial era to >332 nmol mol^−1^ today^2,3^. The primary global source of N_2_O is production during N cycling by microbiota in soils . Soil N cycling also releases NO and N_2_, which directly impact tropospheric ozone production, climate, and soil N content and loss pathways^4,5^. N cycling and thus N gas production are strongly enhanced by direct and indirect anthropogenic N inputs^2,6–8^. Agricultural fertilisation accounts for around two thirds of anthropogenic N inputs^9^, with the remainder contributed by biological N_2_ fixation and deposition of NO_*x*_ and NH_3_. Most anthropogenic N is not incorporated into crops or soils but lost to the environment^10^, representing huge monetary losses for the agricultural sector and causing a cascade of environmental problems^11–13^. In the coming decades, N inputs are expected to grow in line with increasing agricultural production, and to shift towards tropical regions and emerging economies as strict N pollution controls are introduced in many developed regions^14–18^. Effective management of N fertiliser to achieve high N use efficiency is key to balancing food production with environmental protection and thus reducing N_2_O emissions^19,20^. However, mitigation is challenging, and N_2_O emissions are currently exceeding the highest predicted scenarios^2^.

N is lost from terrestrial ecosystems through several major pathways: Microbial and abiotic *N gas production* in soils; runoff and *leaching* of N species; and ammonia *volatilization*. Despite their importance, expected changes in N losses in the coming century are not well known^11,21^. Nitrification (aerobic) and denitrification (anaerobic) are the main processes emitting N gases (NO, N_2_O and N_2_) from soils. The proportion of N inputs released as particular N gases can be described with the emission factor (EF); for example, an EF for N_2_O of 2% means that 2% of annual N inputs are released as N_2_O. On the global scale, the impacts of climate change on N-gas production processes are poorly known: Warming is generally expected to enhance microbial activity and increase N-gas EFs, however interactions between factors such as N availability, plant growth, and precipitation changes are poorly constrained. Moreover, it is unknown if increased nitrification or denitrification rates would in fact lead to increased N gas production^22–24^. The proportion of N lost by leaching globally is not expected to change significantly over time in response to warming due to the contrasting effects of increased N mineralization and reduced moisture availability^2,25–27^. However, leaching losses and predicted responses to warming vary widely between different regions depending on soil, hydrological and ecosystem parameters, and may also be strongly affected by precipitation regime changes^28,29^. Moreoever, increasing atmospheric CO_2_ generally enhances plant growth and N uptake, thus impacting the availability of N in soils for different loss pathways^30,31^. Process models have been used to simulate N loss pathways in a changing climate (e.g.^32–34^). These models used generally require large amounts of input data and parameterisations as well as high computing power, which makes it difficult to iteratively constrain and optimize model parameters with observations using typical inversion frameworks and likelihood approaches, and complicates investigations of global or long-term emissions. Top-down modelling efforts can give robust estimates of global emissions for recent decades^35–37^, however without the incorporation of isotopes, these approaches cannot provide mechanistic information.

The isotopic composition of soil N (*δ*^15^N_soil_) has been proposed as an integrated indicator of N loss partitioning in natural systems^38–41^. Leaching of soluble N species (eg. ${{{{{\rm{NO}}}}}}_{3}^{-}$) involves very low isotopic fractionation, while losses through ammonia volatilization and N gas production strongly favour ^14^N and thus cause ^15^N enrichment in the remaining soil N pool^39^. Observations have shown that mean global *δ*^15^N_soil_ is elevated relative to N inputs, reflecting significant production of gaseous N species^38,39^, although the relationship may be unpredictable in some regions due to N immobilization^41^. Previous studies have used *δ*^15^N_soil_ models to constrain N losses at individual sites or globally for natural ecosystems^38,39^, however this approach has not been applied to estimate temporal changes in N loss pathways. Furthermore, the availability of complementary datasets to validate previous *δ*^15^N_soil_ models has been limited to short-term measurements from individual sites. This approach can be extended to include records of atmospheric N_2_O isotopic composition, which reflects N_2_O production pathways^42–44^, and can be used to validate results from studies of *δ*^15^N_soil_. Atmospheric N_2_O isotopic composition can be described with the N_2_O bulk 15-N isotopic composition (*δ*^15^N^bulk^), hereafter abbreviated as *δ*^15^N, and tje N_2_O 15-N isotopic site preference, hereafter referred to as *δ*^15^N^SP^ (see ref. 45 for definitions and review). Recent advances in spectroscopic isotope instrumentation have delivered high precision long-term time series of background tropospheric N_2_O mixing ratio and isotopic composition^43,45^, allowing an integrated view of N_2_O sources and sinks. These results have been used to estimate total anthropogenic N_2_O emissions based on two-box models of the atmosphere^42,45,46^, however this approach cannot give spatially resolved information on N_2_O sources.

Here we aim to gain new insight into the N loss processes underlying global N_2_O emissions and their spatiotemporal patterns, in order to understand how N losses are changing under the influence of anthropogenic activities and climate change. We use an artificial neural network to estimate a global isoscape of natural soil *δ*^15^N. This is used to initialise a soil module to simulate spatially resolved terrestrial N losses via leaching, volatilization and gas production pathways. N_2_O emissions from the soil module are released to a two-box atmospheric module to simulate N_2_O mixing ratio and isotopic composition from the preindustrial era to the present day. The coupled model framework, ‘IsoTONE’, is optimized within a Bayesian framework using a high precision time series of N_2_O mixing ratio and isotopic composition from several background sites, as well as estimates of N_2_O emission factors from the Global N_2_O Database.

## Results and discussion

### Terrestrial N_2_O emissions

12 key parameters in the IsoTONE model were optimized using a Markov Chain Monte Carlo (MCMC) approach with 120 000 iterations, described in detail in Supplementary Note 3 and Supplementary Table 1. Total terrestrial N_2_O emissions from the optimised model (Table 1) agree well with previous results, providing confidence in the isotopic basis of the model, for example: Total terrestrial N_2_O emissions for 1860, 2010 and 2020 were 5.3 ± 0.4, 12.6 ± 1.2 and 13.9 ± 1.4 Tg N_2_O-N a^−1^ respectively (2020 results shown in Supplementary Fig. 12), showing good agreement with 1860 and 2010 estimates of 6.3 ± 1.1 and 10 ± 2.2 Tg N_2_O-N a^−1^ from the N_2_O Model Intercomparison Project^8^. The range of 12.6 to 13.9 Tg N_2_O-N a^−1^ modelled for 2007-2016 additionally agrees with a recent global meta-analysis estimating total terrestrial emissions of 12.2-23.4 Tg N_2_O-N a^−1^ for the same period^2^, and with the mean of 12.9 Tg N_2_O-N a^−1^ from a meta-analysis by Scheer et al.^47^. Total global soil NO emissions for 2010 were estimated to be 13.7 ± 3.9 Tg NO-N a^−1^, agreeing well with the high end of estimates from a meta-analysis suggesting global soil NO emissions of 1.8-12.3 Tg NO-N a^−1 ^^48^.

**Table 1:** Changing characteristics of the total and anthropogenic terrestrial N_2_O flux at the beginning of the anthropocene (1850) and through the past century

		1850	1940	1980	2020
Total soil flux	Tg N_2_O-N a^−1^	5.3 ± 0.4	6.3 ± 0.5	8.7 ± 0.6	12.4 ± 0.8
Natural soil flux	Tg N_2_O-N a^−1^	5.3 ± 0.4			
Anthropogenic soil emissions	Tg N_2_O-N a^−1^	0	1.0 ± 0.7	3.4 ± 0.7	7.1 ± 0.9
Growth rate of emissions	Gg N_2_O-N a^−1^ a^−1^	19 ± 10	23 ± 0.1	60 ± 5	134 ± 6
Nat. soil emissions—deposition N	Tg N_2_O-N a^−1^	0.5 ± 0.1			
Nat. soil emissions—fixation N	Tg N_2_O-N a^−1^	4.7 ± 0.5			
Anth. soil emissions—fertilisation N	Tg N_2_O-N a^−1^	0	0.1 ± 0.1	0.9 ± 0.2	1.7 ± 0.4
Anth. soil emissions—deposition N	Tg N_2_O-N a^−1^	0	0.7 ± 0.1	1.8 ± 0.2	3.6 ± 0.3
Anth. soil emissions—fixation N	Tg N_2_O-N a^−1^	0	0.2 ± 0.6	0.7 ± 0.7	1.8 ± 0.6
Anth. emissions not from soils	Tg N_2_O-N a^−1^	0	1.0 ± 0.7	1.4 ± 0.7	1.7 ± 0.9
*δ*^15^N, natural soil emissions	‰	−22.4 ± 2.7			
*δ*^15^N, anthrop. soil emissions	‰	NA	−15.3 ± 1.1	−17.0 ± 1.1	−18.7 ± 1.1
*δ*^15^N^SP^, natural soil emissions	‰	6.7 ± 0.6			
*δ*^15^N^SP^, anthrop. soil emissions	‰	NA	5.9 ± 0.2	6.0 ± 0.2	6.8 ± 0.2
EF, area-weighted	%	1.1 ± 0.1			
EF, N input-weighted	%	3.6 ± 0.7	3.8 ± 0.7	4.0 ± 0.8	4.3 ± 0.3

The natural flux and the area-weighted emission factors (EFs) do not vary temporally. Growth rate is the 10-year mean growth rate centred on the year of interest, ie. the 10-year average for 2000 is the average from 1995-2005. The area-weighted EF is the mean global EF calculated using the areas of grid cells as weights; the N-input weighted EF is calculated using the total N inputs of grid cells as weights.

**Fig. 1:**
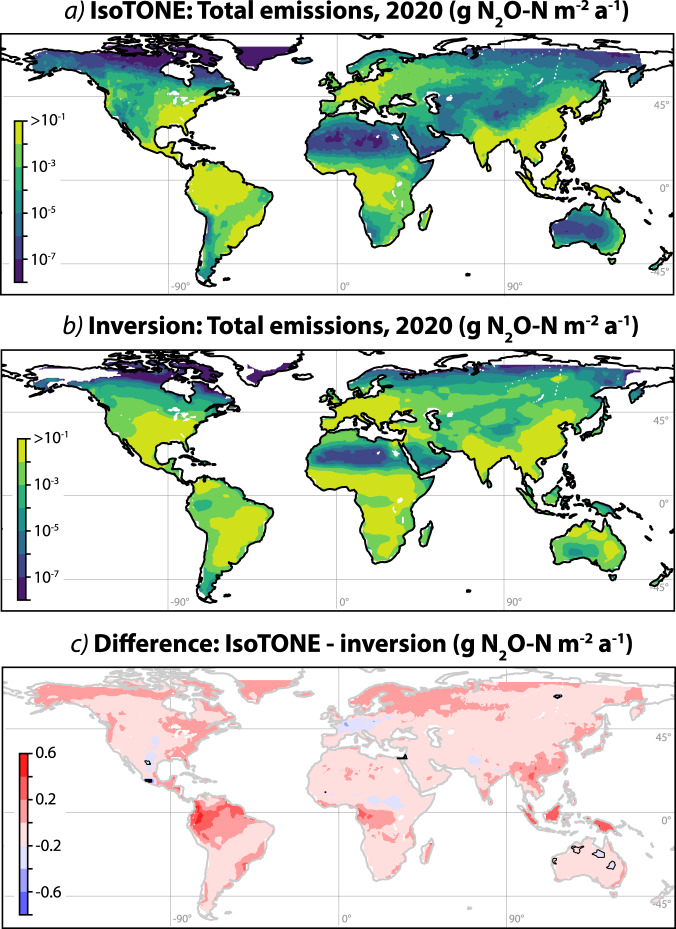
A comparison of total N_2_O emissions from the IsoTONE and CAMS models for the year 2020. Modelled total terrestrial N_2_O fluxes from the IsoTONE model (**a**) and from CAMS (**b**) (R. Thompson, 2021; regridded to 0.5◦using linear interpolation) for the year 2019. Both panels use the same logarithmic colour scale. The difference between the IsoTONE and inversion estimates is shown in **c**; areas where the difference is significant compared to the uncertainty are highlighted with black outlines. Maps generated with Cartopy (Met Office, 2015,^132^).

Natural N_2_O emissions are dominated by N inputs from fixation (4.7 ± 0.8 Tg N_2_O-N a^−1^, 90% of natural emissions). In contrast, anthropogenic soil emissions are dominated by N inputs from deposition (70% of 1940 and 51% of 2020 emissions), although fertilisation is becoming increasingly important (14% of 1940 and 22% of 2020 emissions) (Table 1). The spatial distribution of emissions from fertilisation, deposition and fixation for natural and anthropogenic soils for 2020 is shown in Supplementary Fig. 12, with ‘anthropogenic emissions’ referring to all emissions above the preindustrial baseline, thus accounting for both direct and indirect anthropogenic N_2_O sources. The spatial distribution of N_2_O emissions agrees well with inversion estimates from the Copernicus Atmospheric Monitoring Service (CAMS^49^;) using data from 123 ground-based sites (Fig. 1), with significant differences only seen in small isolated regions. The IsoTONE framework assumes that land use changes—aside from fertiliser use and other N inputs, which are explicitly provided to the model—have had a minor impact on N_2_O emission factors at an annual timescale, compared to the major impact of pre-existing variability in emission factors driven by climate and soil parameters. This assumption is supported by the good agreement between CAMS inversion results and IsoTONE emission estimates. The largest differences are seen in tropical South America, Africa and Australia, which may be due to the scarcity of atmospheric monitoring stations available to constrain inversion estimates in these regions^49^. Differences may also relate to extensive land use change and cultivation of N-fixing soybean crops in South America. Furthermore, fundamental differences in N cycling and immobilization in tropical and Arctic regions may affect the accuracy of the IsoTONE model in these regions, as most experimental and field studies were conducted in temperate soils^40,50^. Total N_2_O emissions agree very well between the two models, with 13.3 ± 0.1 Tg N_2_O-N a^−1^ and 13.9 ± 0.9 Tg N_2_O-N a^−1^ predicted for 2019 by the CAMS inversion and IsoTONE respectively.

The incorporation of isotopic composition means that, unlike previous global models, the IsoTONE framework distinguishes between different N_2_O production pathways (Supplementary Note 4). The model results furthermore suggest that on average, laboratory-measured fractionation factors for these pathways are only expressed at ~55% on average in soils (frac_ex = 0.55 ± 0.05, Supplementary Table 1). This is a global average which we expect to vary widely between individual sites depending on soil structure, moisture, and the depth of N_2_O production and consumption. For example, soils with small N cycling and gas diffusion rates would be expected to have higher isotopic fractionation during N loss processes compared to soils with high diffusivity. In 2020, 60% of N_2_O emitted from soils (7.5 ± 0.4 Tg N_2_O-N a^−1^) was produced from denitrification and 39% (4.7 ± 0.8 Tg N_2_O-N a^−1^) from nitrification. The contribution of nitrification to soil N_2_O emissions has decreased very slightly from 40% in 1850 to 39% in 2020 (Supplementary Fig. 10). The spatial distribution of nitrification-N_2_O from IsoTONE agrees very well with the global map produced by Pan et al.^51^, with high nitrification in areas such as the Amazon basin, sub-Saharan Africa, Europe and coastal Australia. However the model of ref. 51, centers around total nitrification rate, which is not estimated in IsoTONE, so the results cannot be directly compared.

Globally, just 30% of fertiliser N appears to be available for N cycling (fert_EF_red, Supplementary Table 1)—the remaining fertiliser N is primarily incorporated into harvest, and may also be immobilised via increased soil storage, or lost through leaching pathways which are not explicitly modelled. This is consistent with previous meta-analyses, which show that 15–70% of fertiliser N is taken up by plants, a significant proportion could remain in soils, and 5–25% is unaccounted for in plant or soil pools and thus lost to the pathways modelled in this study^10,52–54^. Therefore, emission factors for fertiliser emissions can be significantly lower than for other N inputs, with implications for the applicability of EF measurements from agricultural sites. Mean global fertiliser N incorporation could be increasing as plant growth and thus N use is enhanced in response to increasing atmospheric CO_2_. However, water and nutrient limitations will also play a role in regulating plant growth^2^, and fertiliser use may moreover increase in response to higher N use^55^. These potential effects are not currently captured in IsoTONE and should be a focus of future model studies.

### The anthropogenic N_2_O budget: Inputs, losses and trends

**Fig. 2:**
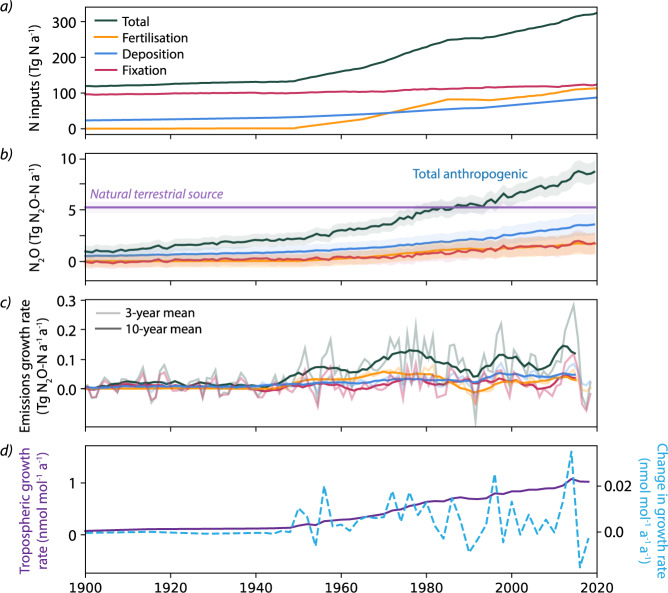
Temporal evolution of N inputs and N_2_O emissions, and growth rates of N_2_O emissions and N_2_O mixing ratio. **a** Annual inputs for fertilisation, deposition, fixation, and total N used in the model (see Methods: at the same geographical locations to gapfill the ancillary data for data sources). **b** The anthropogenic flux broken down into N input categories of fertilisation, deposition and fixation, estimated by assuming that all increases in N_2_O emissions for all input categories (fixation, fertilisation, deposition) after 1850 are due to anthropogenic influences. Total N_2_O emissions, including non-soil N_2_O emissions, are also shown. The shaded areas indicate the 1*σ* uncertainty.N_2_O emission data prior to 1900 as well as the breakdown of natural emissions driven by deposition and fixation are shown in Supplementary Fig. 11. **c** The growth rate of N_2_O emissions from each input category calculated over 3 and 10 year windows (pale and dark lines respectively, using colours indicated for fertilisation, fixation and deposition). **d** The growth rate in modelled N_2_O tropospheric mixing ratio (left axis; purple solid line) as well as the modelled change in mixing ratio growth rate (right axis; blue dotted line).

The anthropogenic N_2_O soil flux in 2020 was estimated to be 7.1 ± 0.6 Tg N_2_O-N a^−1^, close to the highest projected emission scenario (RCP8.5) estimate for 2020^2,56^ (Fig. 2 and Supplementary Fig. 11). 1.7 ± 0.4, 3.6 ± 0.3 and 1.8 ± 0.6 Tg N_2_O-N a^−1^ of the anthropogenic flux were contributed by soil emissions from fertilisation, deposition and fixation N inputs respectively (Table 1), with an additional 1.7 Tg N_2_O-N a^−1^ from non-soil anthropogenic emission sources (emissions from EDGAR for categories 1A1, 1A3b, 2B and 6, see Methods: Atmospheric N_2_O module). This agrees very well with a recent ensemble analysis, which estimated a total anthropogenic flux of 7.3 (4.2–11.4) Tg N_2_O-N a^−1 ^^2^. We find that deposition N accounts for 41 ± 14% of all anthropogenic emissions in 2020 compared to 19 ± 12% direct emissions from fertilisation, 21 ± 15% from enhanced fixation, and 19% from non-soil sources: Deposition N inputs clearly contribute the majority of anthropogenic emissions. This finding is in contrast to the results of Tian et al.^2^, who report that direct N_2_O emissions from fertilisation are dominant. These contrasting results are due in part to the classification of all emissions above the 1850 baseline (eg. enhanced N_2_O from natural sites due to warming, fixation and deposition) being classified as anthropogenic in this study, whereas in previous studies some or all of these processes are not considered in the calculation of the anthropogenic burden. Moreover, our results suggest that emission factors could be significantly underestimated from field measurements (see Sections 1 and 1), likely due to the highly dynamic nature of N_2_O emissions, which are not adequately captured with sparse sampling^20,57,58^. Moreover, measured EFs are often based only on growing season emissions, which can lead to a strong underestimation of annual emissions that could be particularly important in cold regions^59–61^. EFs will be particularly underestimated from field measurements made at agricultural sites, where a significant proportion of N is removed through harvest or immobilization as described by fert_EF_red, thus leading to low EFs. These combined effects will lead to underestimation of deposition emissions when measured EFs are applied at non-agricultural sites in bottom up-N_2_O emission frameworks.

The 10-year mean growth of total anthropogenic N_2_O emissions was between 0 and 0.04 Tg N_2_O-N a^−1^ a^−1^ until ~1945 (Fig. 2). Between 1945 and 1980 the 10-year mean growth rate in emissions increased to 0.12 ± 0.02 Tg N_2_O-N a^−1^ a^−1^ due to rapid growth in fertiliser inputs, after which it stabilised at around 0.1 Tg N_2_O-N a^−1^ a^−1^, corresponding to a steady rate of change of around 0.015 nmol mol^−1^ a^−1^ a^−1^ in tropospheric background N_2_O growth rate. The growth rate of anthropogenic emissions was particularly high between 2010 and 2015 (3-year growth rate up to 0.3 Tg N_2_O-N a^−1^ a^−1^) compared to the average of the last 50 years (0.09 ± 0.06 Tg N_2_O-N a^−1^ a^−1^), in agreement with two recent studies^2,37^. This caused the rate of change for the tropospheric N_2_O burden to peak at 0.026 nmol mol^−1^ a^−1^ a^−1^ (Fig. 2). After 2015, the growth rate of emissions strongly decreased, meaning the 10-year mean growth for 2010–2020 was 0.14 Tg N_2_O-N a^−1^ a^−1^ and thus within then normal range for the last half-century (Fig. 2). Fluctuations in the growth rate for tropospheric background N_2_O mixing ratio reflected the growth rate in emissions driven by different input categories. Variability in fixation N inputs dominates subdecadal interannual variability in total terrestrial N_2_O emissions, such as the 2010–2015 peak, contributing >70% of variability within decadal bins (Fig. 2). Fixation inputs are 2–10 times more variable than other input types at subdecadal timescales, thus accounting for their key role in driving variability in N_2_O emissions. In contrast, changes in fertilisation inputs drive the changing growth rate of anthropogenic emissions at timescales larger than 1–2 decades. Increases in both fertilisation and deposition are responsible for the strong and constant increase in N_2_O emissions and tropospheric background mixing ratio over the last century. These findings could be impacted by uncertainties in the input datasets, which should be investigated further using targeted isotopic and modelling approaches at the site and regional scale in regions with particularly high uncertainty.

The isotopic composition of the total anthropogenic N_2_O source reflects changing emission processes (Supplementary Fig. 11). During the growth rate acceleration of 1945–1980, *δ*^15^N and *δ*^15^N^SP^ of the anthropogenic source were relatively constant, however after 1980 they changed more rapidly. These fluctuations were also observed by Prokopiou et al.^62^, who used a two-box model to interpret N_2_O source isotopic composition from firn air data, and found similar but more uncertain values for anthropogenic source isotopic composition. *δ*^15^N of the anthropogenic source is higher than the mean soil source (Table 1) and shows a strong decreasing trend, which may indicate an increasing dominance of agricultural emissions with low *δ*^15^N-N_2_O after 1980^62^. An increasing proportion of agricultural emissions could also explain the trend in anthropogenic source *δ*^15^N^SP^, which approaches the estimated agricultural mean of 7.2 ± 3.8‰^45^. Other explanations for changing source isotopic composition include changes in the extent of pathways such as consumption via N_2_O reduction and production via nitrification and denitrification. Variability in *δ*^15^N^SP^ and *δ*^15^N are in opposite directions, and thus unlikely to be caused by changes in the extent of N_2_O reduction to N_2_, which would increase both the *δ*^15^N^SP^ and *δ*^15^N of remaining N_2_O^63^. Furthermore, our results suggest a 1% decrease in nitrification contribution to global N_2_O emissions and thus very little change in the nitrification:denitrification ratio, thus this could not account for the observed increase of ~1‰ in *δ*^15^N^SP^ of anthropogenic N_2_O based on *δ*^15^N^SP^ endmembers of 0 and 30‰ for N_2_O from denitrification and nitrification.

### Drivers of spatiotemporal variability in N_2_O emission factors

**Fig. 3:**
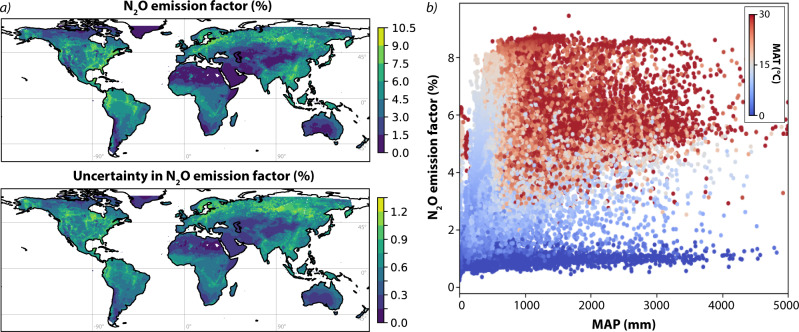
Global map of modelled N_2_O emission factors. **a** Global gridded N_2_O emission factor (upper) and the 1*σ* uncertainty (lower); **b** Relationship between mean annual precipitation (MAP), mean annual temperature (MAT; point colour) and N_2_O emission factor for each grid cell. Maps generated with Cartopy (Met Office, 2015,^132^).

Soil moisture, N content, mean annual precipitation and soil bulk density (21, 9, 5, 4% of variability respectively) were the main parameters causing broad geoclimatic gradients in N_2_O EFs and the proportion of N lost to N gas production between different regions (Fig. 3 and Supplementary Figs. 3 and 13), consistent with previous laboratory and field results^64–67^. Mean annual temperature was important for total N gas production, but not for N_2_O EF (5 vs. 0.2% of variability in *f*_gas_ and EF). Total gas production accounts for the largest proportion of N losses in warm, dry regions, whereas N_2_O EFs are highest in non-desert tropical regions, in particular sub-Saharan Africa, southern India, China, and south east Asia, and low in drier and colder areas. This agrees well with results from refs. 2 and 68, as well as in situ measurement studies showing high N losses via both leaching and denitrification from ‘leaky’ N cycles in tropical regions^69,70^. Geoclimate-driven variability in emission factors can be clearly seen in regions underrepresented in EF compilations, such as Australia, which shows a clear gradient from low to high N_2_O EFs between the dry centre and wetter coastal and northern regions, but very little gradient in total N gas production. This pattern, whereby arid regions show relatively high N gas production dominated by NO, but low N_2_O EFs, can be seen across central North America, the Sahara, Australia, and central Asia. This suggests global applicability of the experimental results from ref. 71, who used isotopic tracing to show high NO production from arid soils was due to reduced plant N uptake and low leaching.

**Fig. 4:**
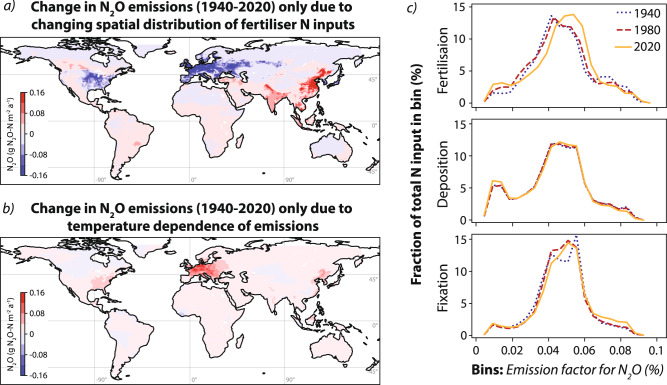
Impact of changing spatial distribution of fertilisation as well as climate warming on N_2_O emissions from 1940 to 2020. **a** The average change in annual N_2_O emissions due to the shifting spatial distribution of fertiliser N inputs, calculated using the 1940 spatial distribution of fertilisation scaled to the 2020 total fertilisation quantity as a baseline, and comparing this to the actual modelled 2020 emissions. **b** The average change in annual N_2_O emissions due to climate warming and the temperature dependency of N_2_O production, found by calculating 2020 emissions with and without the impact of warming on EFs since 1940. **c** The proportion of anthropogenic N inputs from fertilisation, deposition and fixation in 1940, 1980 and 2020 accounted for within bins defined according to the EF for N_2_O. 20 bins were used; 19 were distributed evenly between the parameter minimum and the mean + 3 standard deviations; the highest bin was for all data >mean + 3 standard deviations. Maps generated with Cartopy (Met Office, 2015,^132^).

The spatial distribution of N inputs plays a key role in determining the overall global EF for N_2_O (Fig. 4). The mean global EF for N_2_O weighted by area was 1.1 ± 0.1%, close to the IPCC default value of 1.4% for combined direct and indirect emissions^72,73^. However, the mean global EF weighted by total N inputs per grid cell in 2020 was 4.3 ± 0.3%, as a much greater proportion of total N inputs are in ‘high emission’ temperate and tropical non-arid latitudes compared to dry and cold areas (Fig. 3). This builds upon the observation of Tian et al.^2^, who showed that the mean global EF for N_2_O from agricultural soils is significantly higher than the IPCC default value. Measurements of mean annual EFs using chambers and similar methodologies are far more time intensive and expensive than measurements of soil *δ*^15^N, thus isotopic modelling of the N cycle can be used to understand regional variability in EFs to facilitate upscaling and extrapolation of data from traditional methods. Modelled EFs were compared to the compiled values for croplands used in the meta-anlysis of ref. 74 by finding the mean and standard deviation of all EFs reported by Cui et al.^74^ in each gridcell. Field EF measurements are laborious and challenging, thus relatively few data points are available—of the 55 478 gridcells with a valid modelled EF, only 179 have one or more EF measurements. The agreement between modelled values and observations was relatively good, with a Spearman correlation coefficient of 0.4 (*p* < 0.01) indicating moderate agreement. However, the slope of 0.15 suggested that measurements may consistently underestimate EFs due to insufficient measurement frequency and duration and the importance of ‘hot spots’ and ‘hot moments’ for annual N_2_O emission totals^57,75^. The impact of climate change—in particular changing precipitation patterns—on N_2_O emissions in rapidly developing regions like sub-Saharan Africa and India is not yet captured by observations, and should be a focus of future studies. Understanding the large-scale impact of moisture availability and other climate parameters on N loss processes through targeted measurements campaigns and model development will be key to predicting interactions between climate change-driven precipitation changes and N_2_O emissions.

The proportion of N_2_O lost to different pathways shows clear temporal changes, particularly for fertilisation N inputs, which show an increase in mean EF of N_2_O from <4% prior to 1940 to >5% in 2020 (Supplementary Fig. 14). This has two major causes: Changing spatial distribution of N inputs and climate warming feedback (Fig. 4). The impact of changing spatial distribution of N inputs on annual N_2_O emissions and EFs was estimated by calculating a baseline, using the 1940 spatial distribution of fertilisation scaled to the 2020 total fertilisation quantity, and comparing this to the modelled 2020 maps of emissions and EFs. The shift in fertilisation from North America and other temperate regions (EFs for N_2_O of 4–5%) towards emerging economies in warmer regions with higher EFs (${{{{{\rm{EF}}}}}}_{{N}_{2}O} >$7.5%), in particular China, has caused additional emissions of 0.5 ± 0.3 Tg N_2_O-N a^−1^ since 1940 (Fig. 4c). This effect is only seen for fertilisation N inputs: Deposition and fixation inputs show very minor spatiotemporal changes (Fig. 4c). Deposition inputs are stabilising in China due to vigorous controls on N pollution^76^, and changes in N_2_O from deposition in coming decades are likely to be of minor importance compared to fertilisation- and climate-driven emissions. The impact of climate warming (Fig. 4b) has led to an increase in total microbial gas production of around 3% between 1850 and 2020 (Supplementary Fig. 14), resulting in additional emissions of 0.8 ± 0.4 Tg N_2_O-N a^−1^, and a consequent decrease in leaching losses. The parameterised ‘warming’ impact will be a combination of feedbacks driven by both changing precipitation and increased temperature, which cannot be separated in the current IsoTONE framework. The combined increase of 1.3 Tg N_2_O-N a^−1^ from spatial input changes and climate warming represents 18% of soil and 15% of total anthropogenic N_2_O emissions in 2020. As climate warming continues, and fertiliser use increases in many tropical and subtropical regions, the mean EF of N_2_O and thus the growth rate of tropospheric N_2_O will accelerate, unless significant efforts are focussed on N_2_O mitigation and increased fertiliser N use efficiency in emerging economies^2^. Moreover, the impact of potential unknown climatic feedbacks, in particular non-linear responses associated with extreme events such as drought and flooding, should be a key focus for both measurement and model studies.

### Applications and outlook

N_2_O emissions over the past decades have increased strongly, following the trajectory predicted by the highest emission scenario (RCP8.5)^2^, which suggests an increase in the global emission factor for N_2_O. We developed and applied a coupled soil-atmosphere nitrogen isotope model framework—‘IsoTONE’—using soil *δ*^15^N as an emission proxy to understand the processes underlying spatiotemporal dynamics of global N_2_O emissions. This model set up developed in this study used isotopic composition to trace different N loss and N_2_O production processes, with a Markov Chain Monte Carlo approach implemented to constrain the model using tropospheric time series of N_2_O isotopic composition. Results from IsoTONE agree well with the CAMS inversion model^49^, providing confidence in both methodologies. Compared to CAMS, IsoTONE is able to harness isotopic information to understand spatial variability in emission factors and production and loss processes; moreover, the simplified approach means IsoTONE has low computational requirements and can be used to explore questions requiring many simulations.

The model results show that fixation N inputs drive the majority of natural N_2_O emissions, but deposition N inputs account for the majority of anthropogenic emissions. Fertilisation N inputs are responsible for multidecadal variability in emissions, whereas subdecadal variability in N_2_O emissions is driven by biological N fixation. We show that the effective (N-input weighted) EF in 2020 is 4.3 ± 0.3%, much higher than the IPCC default value of 1.4%. N_2_O EFs are highly spatially heterogeneous—highest in warm, wet ecosystems—and thus strongly underestimated by default values based on area-weighted means. N_2_O EFs have increased over the past century, driven by climate warming as well as spatial redistribution of fertiliser N inputs. These two phenomena have led to additional emissions of 0.8 ± 0.4 and 0.5 ± 0.3 Tg N_2_O-N a^−1^ respectively between 1940 and 2020. Feedbacks between climate warming, spatial changes in agriculture, and N_2_O emissions should be considered in the development of emission projections and mitigation policies. Monitoring of annual N_2_O emissions as well as the soil *δ*^15^N emission proxy in both understudied regions and regions with particularly high emission factors will be key to reduce uncertainty, focus mitigation efforts, and combat rising N_2_O emissions.

## Methods

### Soil, climate and N input datasets

Mean annual surface temperature (MAT) and precipitation (MAP) at 10 minute spatial resolution were taken from the Climate Research Unit high resolution global climatology dataset^77^. Global soil organic carbon estimates and topsoil (0–30 cm) bulk density were taken from the Harmonized World Soil Database with a spatial resolution of 30 arc seconds^78,79^. Aridity index at a spatial resolution of 30 arc seconds was taken from the Global Aridity Index and Potential Evapotranspiration Climate Database (v2)^80^. Global soil pH at >60,000 sites worldwide was taken from the database compiled by Slessarev et al.^81^. The Worldwide Organic Soil Carbon and Nitrogen Dataset^82^ was used to estimate soil organic nitrogen content with data from >4000 sites rasterised using linear interpolation. Total fertiliser N inputs were taken from the Land Use Harmonization Database (LUH) with the historical dataset used for 1800–2015 (LUH2 v2h) and the future forcing dataset for 2015–2020 (LUH2 v2f)^9^. The Community Atmosphere Biosphere Land Exchange (CABLE) Australian community land surface model (^17,83^, https://www.cawcr.gov.au/research/cable/) was used to estimate global mean water-filled pore space (WFPS), fractional NH_3_ losses (no temporal variability) and deposition and fixation N inputs (annual)^17,83,84^. All datasets were converted to the model grid with 0.5^∘^ × 0. 5^∘^ resolution for −180^∘^ to +180^∘^ longitude and −60^∘^ to +85^∘^ latitude (720 × 290 gridcells) using the Python function scipy.interpolate.griddata with linear interpolation.

### Global dataset for *δ*^15^N_soil_

Data for *δ*^15^N_soil_ for >6000 soil samples from natural (non-agricultural) sites was compiled by Craine et al.^41^. This database also includes ancillary data such as MAT, MAP, carbon and nitrogen concentration, C:N ratio, soil texture, density and pH, and site coordinates and elevation, although not all parameters are available for all samples. Geographical coverage was improved by adding a further 748 samples^85–88^, including unpublished data for 112 samples from Australia (BASE database;^89^) and 392 from Africa (experimental sites and set up described in refs. 90–96)—regions which were underrepresented in the Craine database. The majority of data was from near-surface soil (0–50 cm depth). Within this depth range, no significant effect of depth on *δ*^15^N_soil_ was seen in the data. The sample-specific reported ancillary data was compared to the global gridded datasets (see Methods: Soil, climate and N input datasets) at the same geographical locations to gapfill the ancillary data (Supplementary Note 1). Relationships between ancillary data and *δ*^15^N_soil_ measurements were then used to predict *δ*^15^N_soil_ using an artificial neural network (ANN) with the Python package Keras (^97^; details in Supplementary Note 1). Using the ANN, global *δ*^15^N_soil_ on an 0.5^∘^ × 0. 5^∘^ grid was estimated in order to drive the soil isotope module (see Methods: Soil nitrogen module). A bootstrapping approach was used to estimate uncertainty in gridded *δ*^15^N_soil_.

### Atmospheric data

**Fig. 5:**
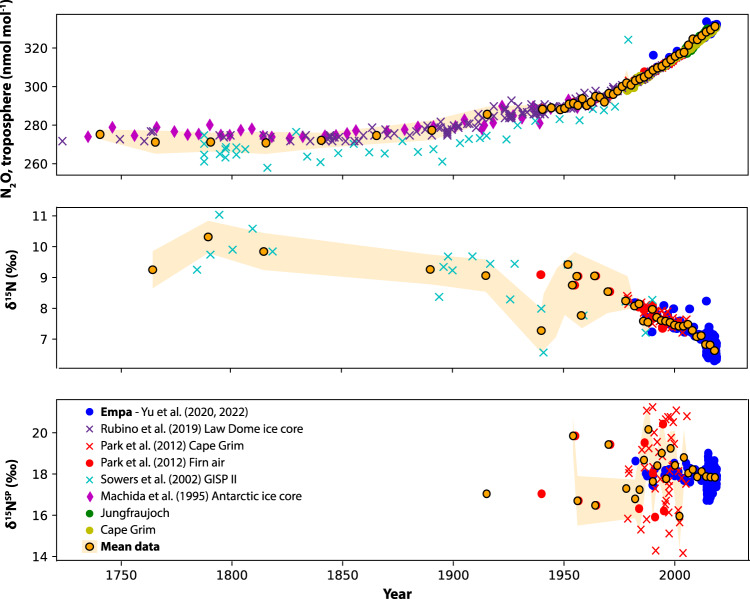
Tropospheric background N_2_O mixing ratio and isotopic composition (*δ*^15^N and *δ*^15^N^SP^) measured in different datasets. Scale offsets between different datasets have been corrected using overlapping periods to match the Empa dataset as described in the text. Mean data at 25-year intervals (1740–1940) and 2-year intervals (1940–2018) are shown as orange points with black outline; the standard deviation of the data averaged for each point is shown as the orange shaded region.

A tropospheric background time series of N_2_O mixing ratio and isotopic composition since the preindustrial era formed the core set of observational data used to constrain simulation results in this study (Fig. 5). The background mixing ratio, for the purposes of this study, is defined as the concentration of a given species when the impact of local or recent sources and sinks is absent—also known as the baseline concentration^98^. The primary dataset comprised tropospheric background measurements of N_2_O mixing ratio, *δ*^15^N and N-isotope *δ*^15^N^SP^ from the Jungfraujoch High Alpine research station and the Cape Grim Air Archive (CGAA), as well as firn air sampled during the 2018 East GReenland Ice coring Project (EGRIP) campaign^45,99^ covering the period from 1982 to the present day, all measured at Empa, Switzerland. Additionally, mixing ratio, *δ*^15^N and *δ*^15^N^SP^ data from CGAA (1979-2005) and firn air (1939-1995) from ref. 6 were used. These were corrected to the Empa dataset scale using the mean offset in the overlap period, with offsets of −1.1‰ and −1.0 ‰found for firn and CGAA data for *δ*^15^N, and +1.5‰ and +1.3‰ for firn and CGAA data for *δ*^15^N^SP^. To extend the isotopic timeseries further back, ice core and interstitial snowpack air measurements from the Greenland Ice Sheet Project II (GISP II)^46^ were also used, covering the period from 1785–1990. This dataset showed no offset for *δ*^15^N compared to the Empa dataset, and did not include *δ*^15^N^SP^ measurements. Other available datasets (e.g.^42^,^62^) were not integrated, as they would not have improved the temporal range of the combined dataset, and would have introduced additional calibration scale uncertainty.

N_2_O mixing ratio in the preindustrial era was constrained using two Antarctic ice core datasets—from ref. 100 covering the period 1735–1964, and from ref. 101 covering the period from 154–1986. Additionally, measurements of N_2_O mixing ratio available at the World Data Centre for Greenhouse Gases (WDCGG, https://gaw.kishour.go.jp) supported by the Global Atmosphere Watch (GAW) Program at Jungfraujoch (2005-present) and the Cape Grim Baseline Atmospheric Pollution Station (1980–present; ALE/GAGE/AGAGE program) were used. Cape Grim data comprise measurements from three instruments covering different time periods, so overlapping periods were used to correct scale offsets, with the most recent dataset (1993–2018) used as an anchor. The dataset from 1981–1994 was +0.03 nmol mol^−1^ offset from the most recent dataset, and the dataset from 1978–1985 was −0.07 nmol mol^−1^ offset from the 1981–1994 dataset.

To allow comparison to simulated results and minimize the impact of any strong episodic local sources, average values for mixing ratio and isotopic composition were binned into 25-year intervals (1740–1940) and 2-year intervals (1940–2018) (Fig. 5). Differences between northern and southern hemisphere sites were not significant given the measurement uncertainty, thus the troposphere was considered as a single well-mixed box to facilitate comparison with IsoTONE simulations. The average standard deviation within each time window for 1740–1940 data was 5.4 nmol mol^−1^, 0.5‰ and 1.2‰ for mixing ratio, *δ*^15^N and *δ*^15^N^SP^, compared to 0.8 nmol mol^−1^, 0.2‰ and 0.6‰ for mixing ratio, *δ*^15^N and *δ*^15^N^SP^ for 2000–2020, reflecting both the strong improvement in measurement techniques for recent in situ data and the particular challenges of firn and ice core measurements. The trend in mixing ratio for averaged data over the last 40 years was +0.8 nmol mol^−1^ a^−1^, with trends of −0.03‰ a^−1^ and −0.006‰ a^−1^ observed for *δ*^15^N and *δ*^15^N^SP^ respectively.

### N_2_O fluxes and emission factors

Data for N_2_O fluxes and emission factors (EF) for >300 sites has been compiled in the ‘Global N_2_O Database’^102^. This database also includes ancillary data such as MAT, MAP, carbon and nitrogen concentration, C:N ratio, soil texture, density and pH, and site coordinates and elevation, although not all parameters are included for all samples. The sample-specific reported ancillary data was compared to the global gridded datasets at the same geographical locations (see Supplementary Note 1). The strongest relationships between ancillary data and emission factors were determined, and used to bin emission factor data into 16 different geographical regions to simplify comparison with modelled emission factors and minimise the impact of localised emission hotspots (see details in Supplementary Note 1: N_2_O emission factors).

### Coupled soil-atmosphere model

**Fig. 6:**
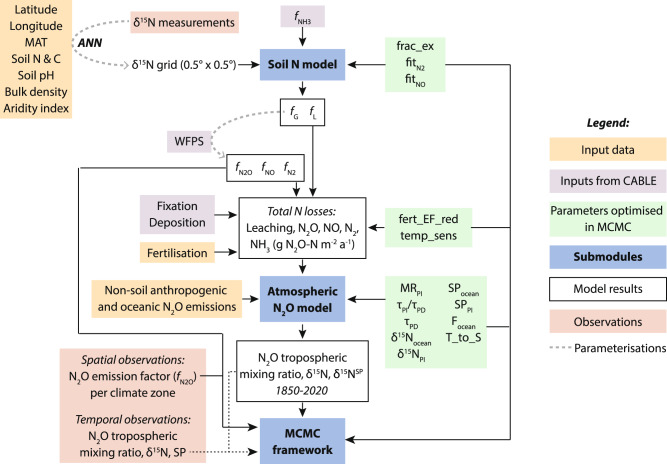
A schematic view of the IsoTONE model and inversion structure, showing different input and output data types. Optimized variables (green) are defined in Supplementary Table 1. Sources of input and observation data are given in the Methods . MAT Mean Annual Temperature, WFPS Water-Filled Pore Space, CABLE Community Atmosphere Biosphere Land Exchange land surface model, MCMC Markov Chain Monte Carlo; *f*_G_, *f*_L_, *f*_NO_, *f*_N2O_, and *f*_N2_ refer to the proportion of N lost through leaching and N gas production, discussed in the Methods.

The model used in this study is an extension of the simple soil isotope model presented by Bai et al.^39^, coupled to an extended version of the atmospheric box model described in ref. 45 to simulate gridded N_2_O fluxes as well as tropospheric mixing ratio and isotopic composition from 1800 to 2020. The full coupled model was written in Python and will be hereafter referred to as the IsoTONE model (ISOtopic Tracing Of Nitrogen in the Environment). A schematic of the model is shown in Fig. 6 and a detailed description is given in the following subsections.

### Soil nitrogen module

The soil N module of IsoTONE estimates fractionational losses of N to different pathways based on *δ*^15^N_soil_ (see Methods: Global dataset for *δ*^15^N_soil_). The module is based on the equations used by Houlton, Bai^38^ and Bai et al.^39^, which state that the *δ*^15^N_soil_ reflects the balance between N inputs (*i*), with a relatively constant rate and isotopic composition, and variable N losses. Loss pathways are divided into leaching (*L*), with low isotopic fractionation; NH_3_ volatilization, with high isotopic fractionation but a low contribution to total losses (*NH*_3_); and abiotic and microbial gas production (*G*), with high and variable isotopic fractionation. At steady state, inputs must equal outputs for both total amounts and isotopic composition:

1 $${f}_{L}+{f}_{NH3}+{f}_{G}=1$$

2 $${\delta }^{15}{{{{{{{{\rm{N}}}}}}}}}_{{{{{{{{\rm{soil}}}}}}}}}={\delta }^{15}{{{{{{{{\rm{N}}}}}}}}}_{i}-{\varepsilon }_{G}\times {f}_{G}-{\varepsilon }_{L}\times {f}_{L}-{\varepsilon }_{NH3}\times {f}_{NH3}$$

where *ε* is the fractionation factor (${k}_{{15}_{{{{{{{{\rm{N}}}}}}}}}}/{k}_{{14}_{{{{{{{{\rm{N}}}}}}}}}}$ expressed in permil, where *k* is the reaction rate) for the respective loss pathway and *δ*^15^N_*i*_ was set as −1.5‰^39^. Assuming steady state, the fraction of N loss accounted for by leaching (*f*_*L*_), NH_3_ volatilization (*f*_*NH*3_) and gas production (*f*_*G*_) can be calculated from *δ*^15^N_soil_. The steady state assumption is only valid for natural ecosystems, thus only *δ*^15^N_soil_ measurements from natural sites were used to initialise the model. Unlike^39^, ^14^N and ^15^N were traced separately in Eq. (2), whereby the rate of ^15^N loss via a pathway is the rate of ^14^N multiplied by the fractionation factor, for example, for leaching:

3 $${k}_{L}^{15}={k}_{L}^{14} \times \left(\frac{{\varepsilon }_{L}}{1000}+1\right)$$

The rate of ^15^N addition was calculated analogously. Tracing isotopes separately facilitated calculation of the stepwise impact of N losses on the remaining N reservoir isotopic composition (‘Rayleigh fractionation’^103^), and necessitated an iterative solution to Eq. (2). Rayleigh fractionation describes how the isotopic composition of reactant and product pools change as a reaction proceeds depending on the fractionation factor and the fraction of reactant consumed, assuming both reactant and product pools are well mixed^103^.

We assumed that N loss processes are primarily linked with the inorganic N pools (i.e. ${{{{{\rm{NO}}}}}}_{3}^{-}$ and ${{{{{\rm{NH}}}}}}_{4}^{+}$), and estimated fractionation factors related to the two species. Fractionation during N mineralization was assumed to be minor^104^. Soil organic N was not considered, given that the rates of turnover processes and their associated isotopic fractionation effects are very scarcely studied at the global scale. Fractionation during leaching (*ε*_*L*_) was estimated to be 1‰^39^. Fractionation during NH_3_ volatilization (*ε*_*NH*3_) was estimated to be −17.9‰ based on the equation in ref. 105. Fractionation during N gas production (*ε*_*G*_) was estimated based on the relative contributions of the two dominant microbial processes: (i) Fractionation for N_2_O production during nitrification of ${{{{{\rm{NH}}}}}}_{4}^{+}$ (*ε*_*nit*_) was estimated as −56.6 ± 7.3‰ with *δ*^15^N^SP^ of 29.9 ± 2.9‰^106^. (ii) Fractionation for N_2_O production during denitrification of ${{{{{\rm{NO}}}}}}_{3}^{-}$ (*ε*_*denit*_) was estimated by adding the fractionation factor for reduction of ${{{{{\rm{NO}}}}}}_{3}^{-}$ to ${{{{{\rm{NO}}}}}}_{2}^{-}$ (−31.3 ± 6.1‰) and for ${rm{NO}}_{2}^{-}$ to N_2_O (−14.9 ± 6.7‰)^106^, thus *ε*_*denit*_ = −46.2‰. *δ*^15^N^SP^ for N_2_O produced from denitrification was estimated as −1.6 ± 3.0‰ (iii) Fractionation for N_2_O reduction during denitrification (*ε*_*red*_) was estimated as −6.6 ± 2.7‰ with *δ*^15^N^SP^ of −5 ± 3‰^106^.

In contrast to bacterial and abiotic denitrification, fungal denitrification results in N_2_O with a high and variable *δ*^15^N^SP^. The partitioning of N_2_O production into fungal denitrification as compared to bacterial nitrification and denitrification (shown in Supplementary Fig. 6) is currently not known and therefore cannot be parameterised within this model. Fungal denitrification is a minor source of N_2_O, expected to contribute less than 10–15% globally^107^. Similarly, co- and chemodenitrification are poorly constrained pathways, both in terms of drivers and isotopic composition^44,108,109^. Fungal denitrification and co- and chemodenitrification will be included in a future version of this model as data to drive parameterisations becomes available.

These fractionation factors refer to the immediate production or consumption of N substrates and N_2_O, and thus do not account for the complexity of processes leading to the net isotopic composition of emitted N_2_O in real environments. Several studies have recognised that soil gas is not a homogeneous, well-mixed environment—instead, gas production and consumption occurs in pores that are only partially connected relative to the rate of microbial processes^110,111^. This means that Rayleigh fractionation processes occur within all pores and microenvironments, and thus the effective net fractionation may be significantly lower than calculated from measured fractionation factors, often referred to as ‘underexpression’^110^. We therefore introduce a parameter called the fractionation expression factor (frac_ex) to the model such that net or effective fractionation = *ε* × frac_ex for all modelled processes. Thereby, frac_ex = 1 would reflect a well-mixed soil gas reservoir where effective fractionation is equal to expectations from laboratory measurements, and frac_ex = 0 would reflect a completely closed soil gas environment where all reactions go to completion within separate pores and thus no effective fractionation is observed. The initial value of frac_ex was set to 0.7 and it was optimized in the MCMC framework (see Methods: Optimization of model N cycle parameters) assuming a uniform error distribution between 0.3 and 1.0. In reality, different values for frac_ex may be expected for different processes and environments, in particular depending on soil texture and pore structure, however this cannot be determined within the available model-data framework.

The soil model was run on an 0. 5^∘^ × 0. 5^∘^ grid from −60^∘^ to 80^∘^ latitude and −180^∘^ to 180^∘^ longitude. The *δ*^15^N grid (see Methods: Global dataset for *δ*^15^N_soil_; Supplementary Fig. 2a) was first initialised in each model run by adding 5% of the uncertainty in *δ*^15^N (Supplementary Fig. 2b) multiplied by a normally distributed random number (*μ* = 0, *σ* = 1). For each grid cell, the soil model was initialised with an available soil nitrogen pool of size 1 (unitless) with *δ*^15^N of 0‰, and a soil N_2_O pool of of size 1 (unitless) with *δ*^15^N and *δ*^15^N^SP^ of 0‰. The ^14^N input rate (*k*_*i*_) was set at 1 (unitless) and the ^15^N input rate (*k*_*i*,15_) calculated using *δ*^15^N_*i*_ of 0.5‰ (based on^39,112–114^). Changing the unitless pool sizes affects only the number of iterations until steady state is reached in the model, and not the final result of the model. Reducing *δ*^15^N_*i*_ by 1‰ increased the calculated mean global *f*_*G*_ by ~1%, while increasing *δ*^15^N_*i*_ by 1‰ increased the calculated mean global *f*_*G*_ by ~5%, with very little impact of spatial distribution of *f*_*G*_.

To solve Eq. (2) for each grid cell, the soil module was run over four iterations (*n* = 4). For the first iteration, *f*_*G*_ was set at 0.1 for all gridcells. *f*_*NH*3_ was estimated using the CABLE model^17,83^. *f*_*NH*3_ is nearly always <0.05, therefore the model results are not highly sensitive to the parameterisation of *f*_*NH*3_. *f*_*L*_ was then determined as 1 − *f*_*G*_ − *f*_*NH*3_. Partitioning of total gas losses (*f*_*G*_) into N_2_O, N_2_ and NO (${f}_{{{{{{{{{\rm{N}}}}}}}}}_{2}{{{{{{{\rm{O}}}}}}}}}$, *f*_NO_, ${f}_{{{{{{{{{\rm{N}}}}}}}}}_{2}}$) was estimated with a sigmoid fit of WFPS to available experimental measurements (Supplementary Note 2, Supplementary Fig. 5), with mean global WFPS from CABLE used to constrain partitioning for each grid cell in the model. Similarly, denitrification (*f*_*denit*_) and nitrification (*f*_*nit*_) contributions to N_2_O production were estimated with a sigmoid fit to available experimental data (Supplementary Note 2, Supplementary Fig. 6). Neither WFPS nor other potential proxies such as soil oxygenation can adequately describe the microenvironment in which microbes conduct N cycling^44,115^. WFPS represents the amount of pore space filled with water and with air and can be compared between soils with different textures, thus it can be used as a proxy for the ability of gases and substrates to move through aqueous and gaseous environments, which is key in determining both substrate supply and soil oxygenation, and thus N cycling and gas production. Mean WFPS will not describe all variability in gas partitioning, given the highly variable and non-linear nature of N gas emissions, however it provides the best available estimate based on the current status of experimental and modelling research. An overall *ε*_*G*_ was estimated based on *f*_*denit*_ and *f*_*nit*_ and the fractionation factors for the individual processes multiplied by frac_ex, with a mean value of −30‰. This is lower than in ref. 39 where a range of −16 to −20‰ was assumed for *ε*_*G*_ without explicit consideration of microbial pathways—measurements made since the publication of ref. 39 have shown that fractionation is much larger than previously estimated^106^. To achieve steady state, 10 cycles of N addition and removal were conducted within each iteration. First, N inputs for ^14^N and ^15^N were added to the initial soil N pool. N was then removed via leaching, volatilization, and gas production for ^14^N and ^15^N. The steady state soil pool *δ*^15^N value at the end of each iteration (*δ*^15^N_*ss*_) was compared to the *δ*^15^N value for the gridcell (*δ*^15^N_*soil*_) to find an improved *f*_*G*_ for the next iteration according to:

4 $${f}_{G,n+1}={f}_{G,n}-\frac{{\delta }^{15}{{{{{{{{\rm{N}}}}}}}}}_{soil}-{\delta }^{15}{{{{{{{{\rm{N}}}}}}}}}_{ss}}{{\varepsilon }_{G}}$$

Following four iterations, *δ*^15^N_*soil*_ and modelled *δ*^15^N_*ss*_ agreed within 0.01‰ and final values of *f*_*G*_ and *f*_*L*_ were accepted.

In addition to the calculation of *f*_*G*_ and *f*_*L*_, the soil module estimated N_2_O production rate and isotopic composition for each grid cell. The production rates for ^14^N-N_2_O and ^15^N-N_2_O were calculated from the fractionation factors for each pathway multiplied by frac_ex; both ^15^N^*α*^ and ^15^N^*β*^ were traced separately to consider N_2_O *δ*^15^N^SP^. For example, the ^14^N reaction rate for nitrification N_2_O production was estimated as ${f}_{G}\times {f}_{{{{{{{{{\rm{N}}}}}}}}}_{2}{{{{{{{\rm{O}}}}}}}}}\times {f}_{nit}$ and the corresponding reaction rate for ^15^N as:

5 $${k}_{15{{{{{{{\rm{N}}}}}}}},nit}={f}_{G}\times {f}_{{{{{{{{{\rm{N}}}}}}}}}_{2}{{{{{{{\rm{O}}}}}}}}}\times {f}_{nit}\times \left(\frac{{\varepsilon }_{nit}\times {{{{{{{\rm{frac}}}}}}}}\_{{{{{{{\rm{ex}}}}}}}}}{1000}+1\right)$$

Analogously, rates for each isotopic variant were also calculated for denitrification production and reduction. The production of N_2_ was assumed to represent the consumption of N_2_O via complete denitrification, as no other significant N_2_ sources are known, thus the extent of N_2_O reduction could be estimated and its isotopic effect calculated. N_2_O production and consumption were estimated in each step of the steady state calculation to give a final N_2_O flux and isotopic composition (*δ*^15^N and *δ*^15^N^SP^) for each gridcell to pass on to the atmospheric module described in the following subsection.

### Atmospheric N_2_O module

The atmospheric module takes the fractional losses, estimated by the soil module described in the previous subsection, and uses a two-box model representing a well-mixed troposphere and stratosphere to reconstruct N_2_O background mixing ratio and isotopic composition. Absolute N emissions into the tropospheric box were calculated using the fractional estimates of losses for each grid cell from the soil module, combined with annual N inputs to each grid cell for fixation, deposition and fertilisation for the period 1800–2020. Fertiliser N inputs were taken from the LUH database and covered the whole time period (see Methods: at the same geographical locations to gapfill the ancillary data). Deposition N inputs for 1860–2050 were from ref. 116. Fixation (for 1901–2100) was estimated using the CABLE model with scenario A1 as described by Peng et al. ^17^, where fixation is calculated using resource optimization with temperature dependence^84^. The 1901 fixation values were used to estimate fixation in earlier years and the 1860 values for deposition in earlier years, thus assuming negligible anthropogenic influence before 1850. As none of the N input datasets included significant anthropogenic inputs before 1850, 1850 is taken as the preindustrial ‘baseline’ throughout this study.

Deposition and fixation inputs were assumed to generally fulfil the steady state criteria required by the soil module, however harvest N exports mean that this assumption is not valid for fertiliser N inputs. Harvested N is often returned to the soil or atmosphere via manure or deposition at a different location^117^. Previous studies have shown that a large proportion of fertiliser N is incorporated into crops and therefore removed during harvest, as well as potentially stored in soils (^10,52,53,117^ and references therein)—thus not available for ‘normal’ N loss partitioning. Therefore, we added a factor fert_EF_red to the model to account for N that is harvested or stored. Fertiliser N inputs are multiplied by fert_EF_red to account for the removal of harvested N or stored in soils before losses by other pathways are calculated; fert_EF_red of 0 would mean that all N is removed in harvest, and fert_EF_red of 1 would mean no N is removed. The isotopic impact of harvest N exports cannot be accounted for within the scope of this study, as the required input data (in particular the proportion of N removed in harvest per grid cell annually) is not currently available. Harvest N will be explicitly incorporated into a future version of the IsoTONE framework. EF changes with fertilisation rate were not considered: A recent meta-analysis showed that the dependence of EF on fertilisation rate is highly variable, and at the global scale EF is not strongly dependent on fertilisation rate^118^, although some studies have shown a strong non-linear relationship between EF and N inputs at the site and regional scale^119^. Tian et al.^2^ report that recent increases in EF for direct soil emissions are likely due to climate change feedbacks and interactions, rather than a direct increase in EF due to increasing fertiliser application. As more evidence regarding the non-linearity of fertiliser EFs becomes available, this will be incorporated into the IsoTONE model.

Previous studies have shown that microbial N gas production is temperature sensitive^23,48,120^ and likely increasing in a warming climate by between 0.5 and 1.0 Tg N_2_O-N ${{{{{{\rm{a}}}}}}^{-1}}^{\circ }$C^−1^, with a best estimate of 0.6 ± 0.2 Tg N_2_O-N ${{{{{{\rm{a}}}}}}^{-1}}^{\circ }$C^−1^^8,33,121^. We assume that this increase in emissions relates to a general sensitivity of microbial activity to temperature, and is therefore likely to affect production of N_2_ and NO as well as N_2_O. We therefore incorporated a temperature sensitivity increase of 10% of microbial N emissions from the year 1800 per degree of warming (temp_sens = 1.1 ± 0.04) for N_2_O, N_2_ and NO, which corresponds to 0.6 Tg N_2_O-N ${{{{{{\rm{a}}}}}}^{-1}}^{\circ }$C^−1^ according to the best estimate of 6.3 ± 1.1 Tg-N a^−1^ for N_2_O emissions in the preindustrial era^8^. Temperature anomalies from the CRUTEM-4.6.0 dataset were used to drive the temperature sensitivity calculation^122^. N losses through leaching were consequently reduced to maintain N mass balance.

Yearly soil emissions of N_2_O, NO and N_2_ were calculated for each input type for each grid cell incorporating both fert_EF_red and temperature sensitivity of emissions. In addition, EDGAR (Emission Database for Global Atmospheric Research,^123,124^) gridded emissions for the major non-soil anthropogenic N_2_O emission categories (1A1 = power industry, 1A3b = road transport, 2B = chemical processes, and 6 = wastewater treatment) were added to estimate total terrestrial N_2_O emissions. Isotopic composition was estimated as *δ*^15^N = 3.9 ± 2.9 and *δ*^15^N^SP^ = 17.6 ± 0.5 for power industry, *δ*^15^N = −7.2 ± 1.2 and *δ*^15^N^SP^ = 10.0 ± 4.3 for road transport, *δ*^15^N = −8.3 ± 10.6 and *δ*^15^N^SP^ = 3.3 ± 5.5 for chemical processes, and *δ*^15^N = −11.6 ± 12.7 and *δ*^15^N^SP^ = 10.0 ± 5.7 for wastewater treatment^43^. Isotopic composition of natural soil N_2_O emissions was given by the soil module for each grid cell, and weighted by the emissions per grid cell to calculate overall isotopic composition of soil N_2_O emissions. The *δ*^15^N isotopic composition of fertiliser N inputs is estimated to be 3‰^125,126^ compared to −1.5‰ for natural N inputs^39^, thus increasing the *δ*^15^N of emitted N_2_O. Total global emissions were found by adding the oceanic emissions (F_ocean_) to the global terrestrial emissions using the flux and isotopic compositions listed in Supplementary Table 1.

Total global N_2_O was emitted into a two-box atmospheric model comprising a well-mixed troposphere and a well-mixed stratosphere, based on the model described in ref. 45. Emissions were assumed to be immediately well-mixed through the atmosphere, as the time required for tropospheric mixing is estimated to be around one year^127^, which is the time resolution of the model. Previous versions of this model explicitly calculate the required preindustrial terrestrial N_2_O flux to achieve steady state in the preindustrial era accounting for best estimates of tropospheric N_2_O mixing ratio, N_2_O lifetime, and stratosphere-troposphere exchange. However, in the atmosphere module of IsoTONE, preindustrial terrestrial emissions (F_terr_) are prescribed by the soil module. An iterative calculation (maximum of 20 iterations) is therefore used to optimize the preindustrial oceanic N_2_O flux (F_ocean_) and the troposphere-stratosphere exchange term (T_to_S) by shifting these two terms stepwise according to their prior uncertainty (Supplementary Table 1) using two equations until steady state is achieved. First, tropospheric N_2_O inputs are balanced against losses to the stratosphere:

6 $${{{{{{{{\rm{F}}}}}}}}}_{{{{{{{{\rm{terr}}}}}}}}}+{{{{{{{{\rm{F}}}}}}}}}_{{{{{{{{\rm{ocean}}}}}}}}}={{{{{{{\rm{T}}}}}}}}\_{{{{{{{\rm{to}}}}}}}}\_{{{{{{{\rm{S}}}}}}}}\times ({{{{{{{{\rm{MR}}}}}}}}}_{{{{{{{{\rm{PI}}}}}}}}}-{{{{{{{{\rm{MR}}}}}}}}}_{{{{{{{{\rm{PI,strat}}}}}}}}})$$

where MR_PI_ and MR_PI,strat_ are the N_2_O mixing ratios in the preindustrial troposphere and stratosphere respectively. The preindustrial stratospheric N_2_O mixing ratio at steady state is then calculated based on stratosphere-troposphere exchange and stratospheric N_2_O destruction^42,63^:

7 $${{{{{{{{\rm{MR}}}}}}}}}_{{{{{{{{\rm{PI,strat}}}}}}}}}=-\frac{{{{{{{{{\rm{MR}}}}}}}}}_{{{{{{{{\rm{PI}}}}}}}}}\times {{{{{{{{\rm{moles}}}}}}}}}_{trop}\times {{{{{{{{\rm{MW}}}}}}}}}_{N2O-N}-{{{{{{{\rm{T}}}}}}}}\_{{{{{{{\rm{to}}}}}}}}\_{{{{{{{\rm{S}}}}}}}}\times {{{{{{{{\rm{MR}}}}}}}}}_{{{{{{{{\rm{PI}}}}}}}}}\times {{{{{{{{\rm{MW}}}}}}}}}_{N2O-N}\times {\tau }_{{{{{{{{\rm{PI}}}}}}}}}}{{{{{{{{\rm{T}}}}}}}}\_{{{{{{{\rm{to}}}}}}}}\_{{{{{{{\rm{S}}}}}}}}\times {{{{{{{{\rm{MW}}}}}}}}}_{N2O-N}\times {\tau }_{{{{{{{{\rm{PI}}}}}}}}}+{{{{{{{{\rm{moles}}}}}}}}}_{strat}\times {{{{{{{{\rm{MW}}}}}}}}}_{N2O-N}}$$

where MW_*N*2*O*−*N*_ is the molecular weight of N in N_2_O (28 g mol^−1^) and moles_*trop*_ and moles_*strat*_ are the moles of air in the troposphere and the stratosphere (1.5 and 0.27 × 10^20^ moles respectively). This iteration meant that optimized values for both F_ocean_ and T_to_S were found for each solution of the model, although they were not explicitly targeted in the inversion described in the following subsection. F_ocean_ was not varied with time, as recent results suggest that the oceanic flux is relatively stable compared to the terrestrial flux^2^. Smaller, dynamic aquatic and semiaquatic ecosystems, such as estuaries and coastal wetlands, show highly dynamic and variably N cycling and N_2_O emissions^128–130^, but are too small to be resolved at the global scale of this study.

Once steady state was achieved for preindustrial N_2_O fluxes and mixing ratio, model calculations proceeded as described by Yu et al.^45^ and will therefore only be briefly presented here. First, net stratospheric fractionation and the isotopic composition of the preindustrial stratosphere were found for *δ*^15^N and *δ*^15^N^SP^ assuming steady state. The model was then run forwards with annual time steps using annual terrestrial emissions and isotopic composition provided by the soil module of IsoTONE. At each time step, N_2_O inputs and destruction were considered to estimate the rate of change in N_2_O mixing ratio and isotopic composition, and thus calculate a time series of mixing ratio, *δ*^15^N and *δ*^15^N^SP^ for a well-mixed troposphere^45^.

### Optimization of model N cycle parameters

Twelve key model parameters were optimised using a Markov Chain Monte Carlo (MCMC) approach (Supplementary Table 1), with several datasets used to constrain the results. The geoclimatic gradients (spatial variability) were constrained using N_2_O emission factors from the Global N_2_O Database, binned for 16 climatic zones (see Methods: N_2_O fluxes and emission factors). Temporal variability was constrained using a combined background tropospheric timeseries of N_2_O mixing ratio and isotopic composition (*δ*^15^N and *δ*^15^N^SP^) from several different sites, averaged for 25-year blocks from 1740 to 1940 and for 2 year blocks from 1940 to 2020 to give a total of 49 data points (see Methods: Atmospheric data and Fig. 5). Data was reduced to 16 climate zone EFs and 49 temporal data points to avoid strong overweighting of recent atmospheric results, which are much more frequent and less uncertain than older measurements (e.g. biweekly monitoring at Jungfraujoch station^45^) but highly covariable. Observation uncertainty was defined as the standard deviation of measurements within each spatial or temporal block. Model uncertainty was set at 0.5 nmol mol^−1^ for N_2_O mixing ratio, 0.1‰ for *δ*^15^N and *δ*^15^N^SP^, and 0.5 for climate zone EF. The incorporation of isotopic composition in the atmospheric model gave an implicit sensitivity to the spatial distribution of emissions, as the isotopic composition of emitted N_2_O depends on the dominant emission processes in a particular gridcell, thus allowing the model to distinguish between changes in different regions and input types.

The MCMC was run with three different stepsizes: 0.75, 0.5 and 0.25. Within each iteration *i* of the MCMC, parameters following a Gaussian uncertainty distribution (see Supplementary Table 1), were varied according to:

8 $${P}_{i+1,G}={P}_{i,G}+1\sigma \,{{{{{{{\rm{uncertainty}}}}}}}}\times {{{{{{{\rm{stepsize}}}}}}}}\times {r}_{{{{{{{{\rm{unif}}}}}}}}}$$

where *P*_*i*,*G*_ is the value of the Gaussian parameter in iteration *i* and *r*_unif_ is a uniformly distributed random number between -1 and 1. Uniform parameters were varied according to:

9 $${P}_{i+1,U}={P}_{i,U}+\frac{{R}_{max}-{R}_{min}}{4}\times {{{{{{{\rm{stepsize}}}}}}}}\times {r}_{{{{{{{{\rm{unif}}}}}}}}}$$

where *P*_*i*,*U*_ is the value of the uniformly-distributed parameter in iteration *i*, and *R*_*max*_ and *R*_*min*_ are the maximum and minimum of the parameter uncertainty range. Independent values of *r*_unif_ were determined for every parameter. Observation uncertainty follows a Gaussian distribution, thus observations were also varied within each iteration using Eq. (8), however *r*_unif_ was determined separately only for different groups of observations, e.g. N_2_O mixing ratio, N_2_O *δ*^15^N^SP^.

Once parameters were varied within an iteration *i*, the Metropolis rule was applied to determine if parameters and observations could be accepted^131^. If both were accepted, the model was run for the parameter set *i*, and the model-observation probability was calculated for *i*. The Metropolis rule was then applied to determine if model-observation *i* could be accepted compared to *i* − 1. 5000 iterations were run at each step size sequentially until a total of 120,000 iterations had been run (40,000 at each step size), which was sufficient to achieve stable results with no significant difference between posterior parameters by step size, and no change in posterior parameters following more iterations. All tested and accepted parameter sets were saved; tested parameter sets were used to check coverage of the parameter uncertainty space (Supplementary Fig. 7), and accepted parameter sets were used to find posterior results and uncertainties for the parameters (Supplementary Table 1). The parameters F_ocean_ and T_to_S (see Methods: Atmospheric N_2_O module) were not explicitly varied in the MCMC but were calculated to achieve steady state in the atmospheric module in each accepted iteration, thus posterior estimates for these parameters were also obtained.

### Estimating uncertainty in posterior model results

The uncertainty in posterior parameters shown in Supplementary Table 1 was estimated as the standard deviation of all accepted results. To estimate uncertainty in the posterior model results, 100 iterations of the model were run using 100 randomly selected sets of accepted parameters. The standard deviation of results from all 100 iterations was used to estimate the uncertainty in the final model results. Standard error propagation was used to estimate uncertainty in all subsequently calculated values, eg. ratios and sums across time or space.

## Supplementary information

Supplementary Information

Peer Review File

## Data Availability

The gridded input datasets generated in this study have been deposited in the public, open access model code repository: https://github.com/elizaharris/IsoTONE. *δ*^15^N_soil_ point data not present in the^41^ dataset are also included in this repository, and also archived in the Pangaea data repository (doi currently being processed). Tropospheric background N_2_O isotopic data collected at Empa and used for model optimization will be released open access in 2022 together with the associated manuscript^99^.

## Code availability

The full IsoTONE model code including implementation of the MCMC optimization is included in the public, open access repository: https://github.com/elizaharris/IsoTONE. The repository doi for the associated release IsoTONE_v1 is 10.5281/zenodo.6772207.
