# Supplementary material for: Warming and redistribution of nitrogen inputs drive an increase in terrestrial nitrous oxide emission factor
Source: Nat Commun. 2022 Jul 25;13:4310. doi: 10.1038/s41467-022-32001-z (PMC9314393; doi:10.1038/s41467-022-32001-z)
Supplement: Supplementary file 1 — Supplementary Information [file 41467_2022_32001_MOESM1_ESM.pdf]

Supplementary information for:
Warming and redistribution of nitrogen inputs drive an increase in terrestrial nitrous oxide emission factor

E. Harris*[1,2], L. Yu*[3,4], Y-P. Wang[5], J. Mohn[4], S. Henne[4], E. Bai[6], M. Barthel[7], M. Bauters[8], P. Boeckx[8], C. Dorich[9], M. Farrell[10], P. B. Krummel[5], Z. M. Loh[5], M. Reichstein[11], J. Six[7], M. Steinbacher[4], N. S. Wells[12,13], M. Bahn[2], and P. Rayner[14,15]

[1]Swiss Data Science Centre, ETH Zurich, 8092 Zurich, Switzerland
[2]Functional Ecology Research Group, Institute of Ecology, University of Innsbruck, 6020 Innsbruck, Austria
[3]Institute of Environment and Ecology, Tsinghua Shenzhen International Graduate School (SIGS), Tsinghua University, Shenzhen 518055, China
[4]Laboratory for Air Pollution & Environmental Technology, Empa, Swiss Federal Laboratories for Materials Science and Technology, 8600 Duebendorf, Switzerland
[5]Climate Science Centre, CSIRO Oceans and Atmosphere, Aspendale, VIC 3195, Australia
[6]Key Laboratory of Geographical Processes and Ecological Security of Changbai Mountains, Ministry of Education, School of Geographical Sciences, Northeast Normal University, Changchun 130024, China
[7]Department of Environmental Systems Science, ETH Zurich, 8092 Zurich, Switzerland
[8]Isotope Bioscience Laboratory – ISOFYS, Department of Green Chemistry and Technology, Ghent University, Coupure Links 653, 9000 Ghent, Belgium
[9]Natural Resource Ecology Laboratory, Colorado State University, Fort Collins 80523, CO, USA
[10]CSIRO Agriculture and Food, Locked bag 2, Glen Osmond, SA 5064, Australia
[11]Department of Biogeochemical Integration, Max Planck Institute for Biogeochemistry, Jena, Germany
[12]Centre for Coastal Biogeochemistry, Southern Cross University, Lismore, NSW 2480, Australia
[13]Department of Soil and Physical Sciences, Agriculture and Life Sciences, Lincoln University, Lincoln 7647, New Zealand
[14]School of Geography, Earth and Atmospheric Sciences, University of Melbourne, Parkville, VIC 3052, Australia
[15]Melbourne Climate Futures Climate and Energy College, University of Melbourne, Parkville, VIC 3052, Australia

Corresponding author: eliza.harris@sdsc.ethz.ch
*E. Harris and L. Yu contributed equally to this manuscript.

Contents

Supplementary Note 1: Geoclimatic gradients in $\delta^{15}N_{soil}$ and in N_2O emission factors **3**
Gradients in $\delta^{15}N_{soil}$ 3
Gradients in N_2O emission factors 6

Supplementary Note 2: Parameterisation of N gas production pathways **8**

Supplementary Note 3: Model optimization **10**

Supplementary Note 4: Partitioning N_2O emissions as nitrification and denitrification **16**

Supplementary Note 5: Estimation of anthropogenic N_2O flux and isotopic composition **17**

Supplementary figures 12-14 **19**

Supplementary Note 1: Geoclimatic gradients in $\delta^{15}N_{soil}$ and in N_2O emission factors

Gradients in $\delta^{15}N_{soil}$

$\delta^{15}N_{soil}$ data from >7000 samples from natural (non-agricultural) sites was compiled as described in the Methods, however ancillary data for these measurements (e.g. MAT, MAP, pH) was incomplete. Ancillary data reported for the point measurements was therefore compared to the global gridded datasets (see Methods). Mean annual temperature (R^2=0.96, slope=0.98), precipitation (R^2=0.87, slope=0.67) and pH (R^2=0.60, slope=0.62) agreed well between the point and gridded datasets. Soil carbon showed moderate agreement (R^2=0.3, slope=0.35), while soil N and C:N ratio showed no correlation between the point and gridded datasets. The relatively poor agreement for soil C and N can be attributed to the high spatial variability of these parameters; additionally, the gridded datasets refer to organic C and N while the point data does not always report whether organic or total C and N were measured. A complete ancillary dataset was compiled by gapfilling missing point values of MAP, MAT and pH with gridded dataset values. For C, N and C:N only gridded values were used, as point and gridded datasets were inconsistent and point values are likely less comparable between sites. Other relevant parameters, such as bulk density, which were not reported or very sparsely reported in the point measurements were taken from the gridded datasets.

An overview of the relationships between ancillary data and $\delta^{15}N_{soil}$ was gained using single linear correlation analyses and a multivariate principal components analysis (Supplementary Fig. 1). The strongest positive linear correlations were found between $\delta^{15}N_{soil}$ and MAT, N and bulk density, and the strongest negative correlations between $\delta^{15}N_{soil}$ and C, C:N, elevation and aridity index. Only parameters with <1000 missing values were used for the principal components analysis, therefore elevation and soil texture were not included. PC1 was dominated by the broad geoclimatic gradient between wetter and drier areas (based on MAP), which did not relate strongly to $\delta^{15}N_{soil}$, in contrast to previous expectations [1]. PC2 (24.6% of variability) showed a relationship between MAT, N, C, C:N and $\delta^{15}N_{soil}$ evident from the single linear correlations, thus reflecting the impact of temperature on soil chemistry and N cycling. PC3 (12.4% of variability) showed a relationship between $\delta^{15}N_{soil}$, C, N, C:N and bulk density and aridity, and therefore a weaker relationship between water availability and soil chemistry. Similar to the results of [19] describing the spatial variability of soil organic carbon, climate was key in driving variability in $\delta^{15}N_{soil}$ of near-surface layers, and physicochemical properties were secondary but nonetheless important.

The $\delta^{15}N_{soil}$ data is not globally representative and many large regions have very sparse data coverage, thus interpolation of the data to a global grid is not possible. An artificial neural network (ANN) was therefore used to predict $\delta^{15}N_{soil}$ based on geoclimate parameters and thus estimate a global grid of $\delta^{15}N_{soil}$ values using the 'Keras' package [6] in Python. A sequential ANN was compiled with:

1. 9 input nodes;
2. A dense layer with 16 nodes using the rectified linear unit (ReLU) activation function;
3. A dense layer with 16 nodes using a hyperbolic tangent (tanh) activation function, and L2

regularization with a regularization parameter of 0.01;

4. Identical to layer 3. but using a linear activation function;

5. A batch normalization layer;

6. A dense output layer with one node using a ReLU activation function.

Weights were initialised with Xavier initialization using a uniform distribution (default initialization in Keras). The Adam optimizer was used with a learning rate of 0.0005 and root mean squared error (RMSE) as a loss function. During training, a validation split of 25% was used to minimise overfitting, and early stopping was implemented based on the minimum loss function for validation data with a patience level of 30 epochs. A model checkpoint was used to save the model that achieved the lowest RMSE for the validation data during the training period. Training was carried out over a maximum of 80 epochs with a batch size of 200, and data shuffling was activated. As samples were not distributed evenly across different regions, sample weighting to reduce the relative importance of samples from highly represented regions such as Europe was needed. Samples were grouped into 30 clusters according to latitude and longitude using the KMeans function from the 'sklearn' package. A sample weight vector was calculated as $\frac{1}{\sqrt{n}}$ where n is the number of members in a cluster. The sample weights were used to weight the loss function during the ANN training.

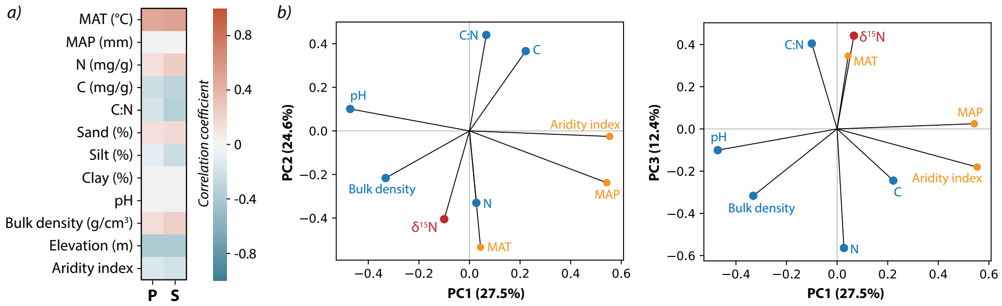

Supplementary Figure 1: Relationships between $\delta^{15}N_{soil}$ and ancillary data. *a*) Color map showing linear correlation coefficients (P = Pearson, S = Spearman) indicated with the color bar between $\delta^{15}N_{soil}$ and the specified parameter. *b*) Principal components analysis of the combined $\delta^{15}N_{soil}$ and ancillary data; soil parameters are shown in blue and climate parameters in orange.

A bootstrapping approach was used to find the ANN model best able to predict the observation data and to estimate the uncertainty in predicted $\delta^{15}N_{soil}$. First, the relevant uncertainty in the $\delta^{15}N_{soil}$ measurements was determined; this relates primarily to the degree to which a single measurement is representative of soils in a grid cell, and is much higher than the measurement uncertainty (usually <0.2‰ for IRMS measurements). The representation uncertainty was estimated to be 1.2‰, based on the mean of the standard deviation of all measurements placed within each grid cell for the 461 grid cells with more than 2 measurements. 250 bootstrap iterations were run, and within each iteration, data for $\delta^{15}N_{soil}$ was perturbed according to the representation uncertainty multiplied by normally distributed random numbers. $\delta^{15}N_{soil}$ and the chosen input parameters (latitude, absolute longitude, MAP, MAT, soil N, soil C, soil C:N, pH, bulk density, and aridity index) were randomly split into training ($\frac{2}{3}$ of data) and testing subsets. Data was normalised to a range of 0-1 for each parameter, and all rows containing NAs were removed.

6000 rows of data were used for training and 3000 for testing the ANN in each iteration. The trained ANN was used to predict the test dataset values of $\delta^{15}N_{soil}$. The predicted and observed test $\delta^{15}N_{soil}$ were compared using the weighted root mean square error (wRMSE) and the R^2 of a weighted linear fit within each bootstrap iteration, using $\frac{1}{\sqrt{n}}$ where n is the number of members in the k-means cluster size as the weight for each point. The ANN achieving the best fit (lowest wRMSE and highest R^2) on the test data was used for the final prediction of $\delta^{15}N_{soil}$, and the standard deviation of the predicted $\delta^{15}N_{soil}$ from all bootstrap iterations was used to estimate the uncertainty.

The final ANN was able to reproduce the observations with a slope of 1.04, R^2 of 0.41 and wRMSE of 2.6‰, thus exhibiting significantly more explanatory power than a multiple linear regression, which was only able to reproduce data with R^2 of 0.27 and RMSE of 3.1‰. The role of specific parameters in the ANN was examined using two methods: i) using the ANN to predict $\delta^{15}N_{soil}$ with all inputs set to their mean values, except for the parameter of interest, to understand how much of the variability in $\delta^{15}N_{soil}$ is contributed by the parameter, and ii) shuffling all data for the parameter of interest before predicting $\delta^{15}N_{soil}$, to measure the reduction in goodness-of-fit. MAT was the most important parameter in the ANN, accounting for 30% of variability in $\delta^{15}N$ and an RMSE reduction of 0.8‰, followed by absolute longitude (14.5%, 0.3‰), latitude (9.5%, 0.2‰), pH (9.8%, 0.07‰) and bulk density (8.7%, 0.16‰). The importance of MAT as well as soil composition agrees with previous studies by [1] and [8].

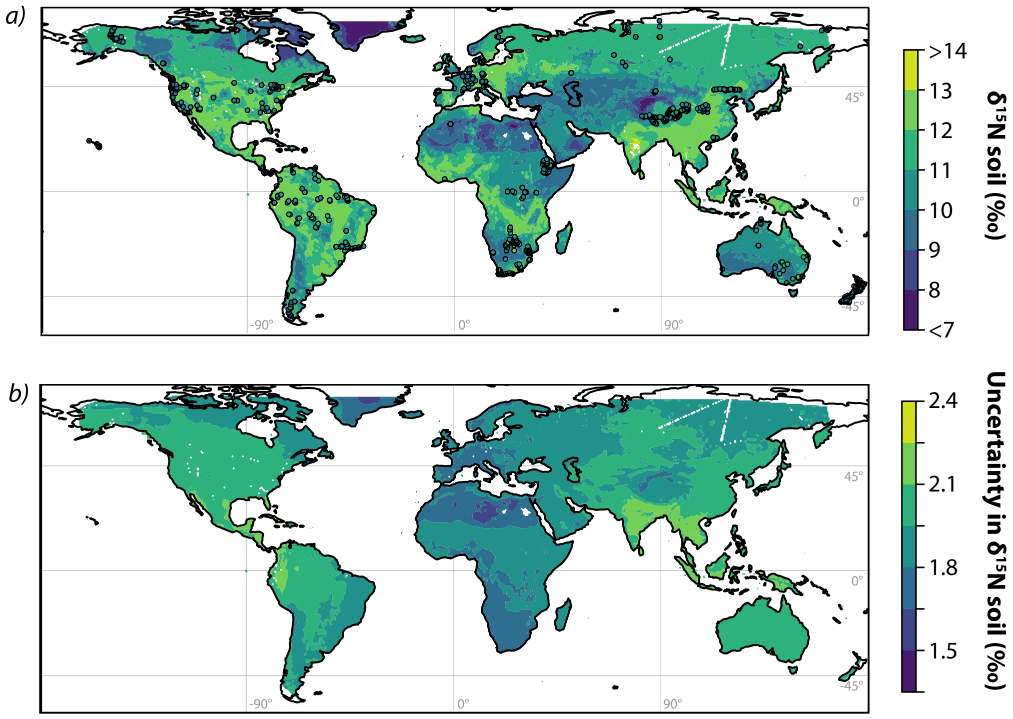

Supplementary Figure 2: *a*) Global gridded (0.5° × 0.5° grid) $\delta^{15}N_{soil}$ estimated using an artificial neural network. Note the colour scale is non-linear to better show the narrow range of most values. *b*) Estimated uncertainty in gridded $\delta^{15}N_{soil}$. Maps generated with Cartopy (Met Office, 2015, [20]).

Gradients in N_2O emission factors

2 793 measurements of N_2O fluxes and 3 629 EFs from 336 locations were taken from the 'Global N_2O Database' [11], and used to examine variability in fluxes and EFs along geoclimatic gradients. Ancillary data was not provided for all measurement points, therefore as described in Supplementary Sect. for $\delta^{15}N_{soil}$ data, incomplete ancillary data was filled in with the global gridded datasets for the same geographical locations. As described in the previous subsection, agreement was very good for well-defined parameters like MAT and MAP, and relatively poor for heterogeneous or variable parameters like soil N content. Linear correlations and PCA for the combined flux and EF dataset revealed the parameters linked most strongly to N_2O emissions - particularly aridity, pH, soil C, MAT and MAP (Supplementary Fig. 3).

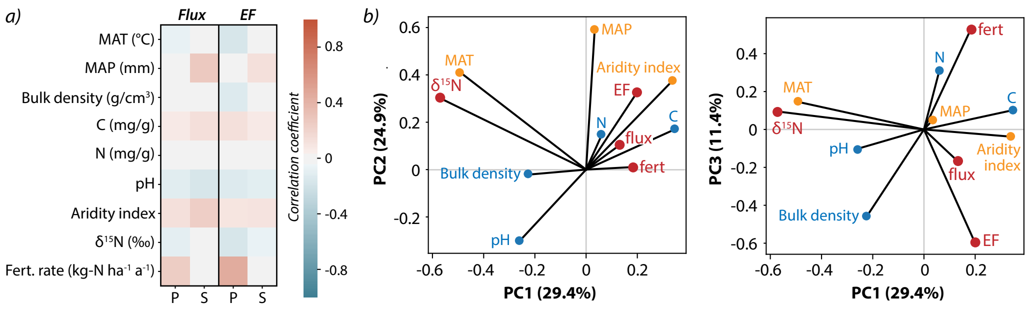

Supplementary Figure 3: Relationships between N_2O fluxes and emission factors (EFs) and ancillary data. *a*) Color map showing linear correlation coefficients (P = Pearson, S = Spearman) for fluxes and EFs indicated with the color bar. *b*) Principal components analysis of the combined soil flux, EF and ancillary data; soil parameters are shown in blue, climate parameters in orange, and N-cycle parameters in red.

N_2O EFs are predicted to be highest at low pH, consistent with the observations from the 'Global N_2O Database' [15, 17]. Both water availability and bulk density impact O_2 availability, which is known to be key in determining nitrification and denitrification rates and thus N gas emissions [21, 15]. The observations are inconclusive regarding the impact of soil N availability on EF. The correlation analysis shows a relationship between fertilisation and EF only for the Pearson correlation coefficient, which is potentially more affected by outliers, but no relationship between EF and soil N availability. Soil N and fertilisation do not plot strongly against PC1 or PC2; fertilisation shows a strong positive relationship and soil N availability shows a weak negative relationship to EF along PC3. The lack of a strong relationship between soil N inputs and availability and EF may also be due to sub-grid cell processes and strong heterogeneity in soil nitrogen, which cannot be captured on the coarse spatial scale currently used in the IsoTONE framework. Our results are consistent with numerous studies, which show that although more N availability clearly leads to more N gas production, the relationship between N availability and N gas EFs is unclear [4, 17, 31, 9]. [32] report increasing EF for direct soil emissions over recent decades that is attributed to climate change feedbacks and interactions as well as spatiotemporal variability of climate, rather than a direct increase in EF due to increasing fertiliser application.

In order to compare modelled EFs to the EF dataset, which came from a range of agricultural ecosystem types with varying management practices, measurement methods and time periods, it was necessary to estimate mean EFs for different 'climate zones'. Although aridity index (AI)

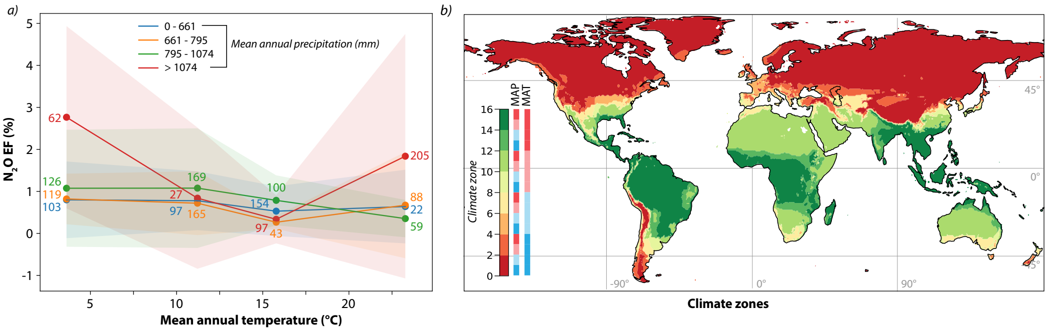

Supplementary Figure 4: Mean emission factors for different climate zones based on measured EF data binned according to MAT and MAP. *a*) Mean EFs for the different climate zones; MAT (mid-point of bin) is plotted on the x-axis and line colours refer to the MAP bin. The standard deviation of values in each bin is shown with the shaded region and the number of values in each bin is annotated. *b*) Global map showing the spatial coverage of the 16 bins: MAT increases with bin number, and MAP increases sequentially through bins 1-4, 5-8, 9-12 and 13-16. Maps generated with Cartopy (Met Office, 2015, [20]).

and soil C content correlated strongly with EF, there was strong non-linearity in these parameters, as well as limited variability within the dataset because the sites with EF data are strongly biased towards northern hemisphere temperate regions. Therefore, MAT and MAP were selected as the optimal parameters to define climate zones to bin EFs, based on both correlations with fluxes and EFs as well as good coverage ranges for available data within each bin. The quartile ranges for MAT and MAP were used to define 16 climate zones for which mean EFs could be calculated (Supplementary Fig. 4). Standard deviation within each climate zone bin was high (Supplementary Fig. 4), reflecting the high spatial and temporal variability of N_2O fluxes as well as the lack of robust annual flux monitoring in many locations. Due to the high uncertainty, no clear relationship between EFs and MAT or EFs and MAP was seen - except for sites with MAP $>$1074 mm a^{-1}, which had signficantly higher EF at both low and high MAT. Hot spots and moments mean that N_2O annual fluxes and EFs are challenging to measure and highly uncertain, often depending on methodological factors such as the number of replicates, spatial replication, and temporal frequency [39, 14, 13, 28]. The uncertain EF data will therefore place little constraint on the model, however the estimates of posterior EFs for the different climate zones will be useful to evaluate the accuracy of the measurement data and reduce uncertainty in estimates of EF in different regions.

Supplementary Note 2: Parameterisation of N gas production pathways

Production of N_2O, NO and N_2 as a proportion of total N gas production as well as the proportion of N_2O contributed by the nitrification and denitrification pathways were parameterised as a function of WFPS using experimental data (including all data used by [3] as well as additional data from [40, 16, 38, 35, 12]). Experimental data was only available for $N_2O/(N_2O + N_2)$ and $N_2O/(N_2O + NO)$ and not for all three gases simultaneously, therefore each dataset was fit separately with a sigmoid curve using the `curve_fit` function from the `scipy` package (Supplementary Fig. 5). Data covered a range of ecosystem types including forest, native and managed grassland, tropical forest, pasture and rice paddy (data used by [3] and additional data from [40, 16, 38, 35, 12]). The proportion of each gas of the total N gas production ($N_2O + N_2 + NO$) was found by combining

the two sigmoid fits:

$$\frac{\mathrm{N_2O}}{\mathrm{N_2O}+\mathrm{NO}} = a = \frac{1.2}{1+e^{-0.04\times(\mathrm{WFPS}-\mathbf{66.5})}} \tag{1}$$

$$\frac{\mathrm{N_2O}}{\mathrm{N_2O}+\mathrm{N_2}} = b = \frac{0.76}{1+e^{0.07\times(\mathrm{WFPS}-\mathbf{38.7})}} + 0.32 \tag{2}$$

$$\frac{\mathrm{N_2O}}{\mathrm{N_2O}+\mathrm{N_2}+\mathrm{NO}} = \frac{1}{\frac{1-a}{a}+\frac{1-b}{b}+1} \tag{3}$$

Two model parameters ($\mathrm{fit_{N2}}$ and $\mathrm{fit_{NO}}$) represented in the MCMC were used to optimize the parameterisation of N_2O, NO and N_2 as a fraction of total N gas emissions, by varying the most sensitive parameter in each sigmoid fit to cover the spread of the experimental data (parameter highlighted in red bold in the above equations; fits shown in Supplementary Fig. 5). The optimized fit shows that NO dominates emissions at low WFPS and N_2 at high WFPS, while N_2O is emitted across the whole WFPS range. The contribution of N_2O steadily increases from $\sim$1% at 0% WFPS to $\sim$32% for WFPS $>$60%. Compared to the empirical fit made in [3], N_2O remains higher and N_2 lower in very wet soils for both the prior and posterior. The posterior parameterisation is described by:

$$\frac{\mathrm{N_2O}}{\mathrm{N_2O}+\mathrm{NO}} = a = \frac{1.2}{1+e^{-0.04\times(\mathrm{WFPS}-\mathbf{81.3})}} \tag{4}$$

$$\frac{\mathrm{N_2O}}{\mathrm{N_2O}+\mathrm{N_2}} = b = \frac{0.76}{1+e^{0.07\times(\mathrm{WFPS}-\mathbf{36.5})}} + 0.32 \tag{5}$$

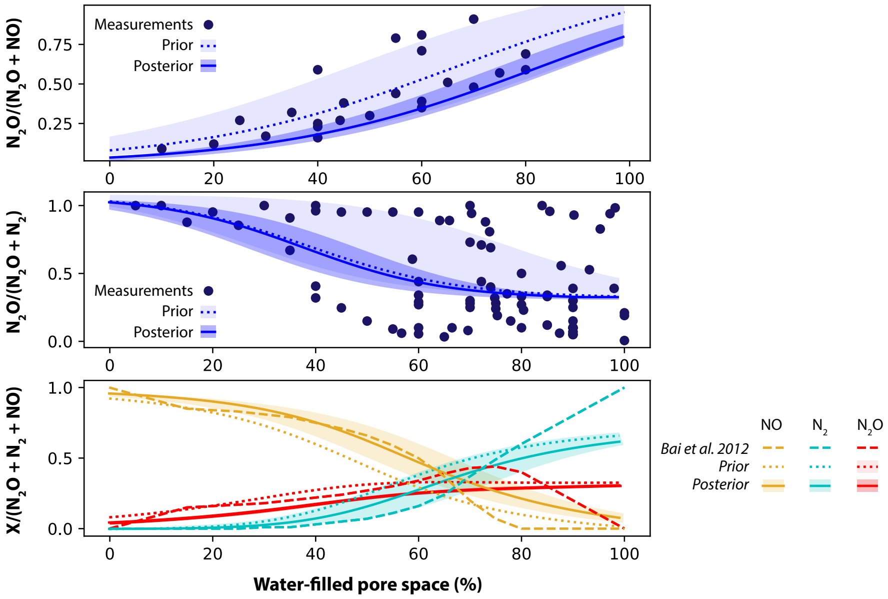

Supplementary Figure 5: Parameterisation of N_2O/NO/N_2 as a fraction of total N gas emissions based on water-filled pore space. The upper panels show experimental data; prior estimates of the parameterisation using sigmoid fits to the data are shown as dotted lines, with the prior 1σ uncertainty range shown with pale shading. Posterior fits following the MCMC optimization are shown as solid lines with the posterior 1σ uncertainty range in darker shading. Eqs. 1, 2 and 3 describe the prior and Eqs. 3, 5 and 4 the final fits. The bottom panel shows the proportion of total N gas contributed by NO, N_2O and NO according to the parameterisation used by Bai et al. 2012 (dashed line), the prior fits (dotted line) and the posterior fits (solid line with uncertainty range shaded).

Experimental data to constrain the relative contributions of nitrification and denitrification to N_2O emissions is sparse; only 45 measurements were available, from grassland [16, 38, 12] and rice paddy [35] systems. Although the data was not representative of global ecosystem types, a wide range of WFPS was covered. A sigmoid curve fit the data well (Supplementary Fig. 6):

$$\frac{N_2O_{\text{denit}}}{N_2O} = \frac{0.2}{1 + e^{-1.5\times(\text{WFPS}-59.7)}} + 0.23 \tag{6}$$

Unexpectedly, denitrification makes a slightly lower contribution to N_2O emissions at higher WFPS, because a significant amount of N_2O is reduced to N_2 before emission at high WFPS. However, the scarcity of data means that this parameterisation is highly uncertain, and further laboratory and field measurements of the partitioning between pathways are needed to better constrain the roles of nitrification and denitrification. Furthermore, the denitrification to nitrification ratio is likely also dependent on agricultural management and fertiliser application, estimation of which is currently beyond the scope of this model. Recent developments in spectroscopic isotope measurements offer a promising method for online, *in situ* quantification of nitrification and denitrification N_2O emissions [41, 12], which offers the potential for improvements to the parameterisation as new data from a wider range of sites becomes available.

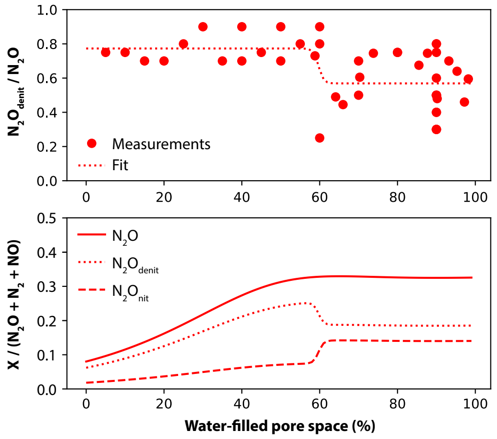

Supplementary Figure 6: Parameterisation of denitrification and nitrification contribution to N_2O emissions based on water-filled pore space. The upper panel shows experimental data for denitrification N_2O as a proportion of total N_2O emissions with a sigmoid fit to the data. The lower panel shows N_2O, N_2O_{denit} (dotted line) and N_2O_{nit} (dashed line) as a proportion of total N gas emissions using the posterior fit described in Eqs. 3, 4, 5 and 6.

Supplementary Note 3: Model optimization

120 000 iterations of the MCMC framework were run and 1 667 solutions were found. 40 000 iterations were run for each step size 0.25, 0.5 and 0.75, with 1 760 (4.4%), 76 (0.2%) and 34 (0.1%) solutions accepted respectively (Supplementary Fig. 7 and Supplementary Table 1). There were no significant differences in the means of accepted values for the different stepsizes, and no change in the final values if only a subset of the accepted values randomly sampled, showing that the posterior parameters were robust. The posterior parameters showed a strong reduction in uncertainty compared to the prior for most parameters (Supplementary Fig. 7 and Supplementary

Table 1), although τ_{PI}/τ_{PD}, τ_{PD} and temp_sens - which are all primarily constrained by N_2O mixing ratio increase - cannot be easily separated during model-data assimilation. F_{ocean}, τ_{PD} and T_to_S showed the strongest correlations between accepted solutions and dominated PC1 in the PCA (Supplementary Fig. 8), again reflecting their role in regulating N_2O mixing ratio increase in the atmosphere submodule. Addition of an independent tracer of T_to_S, or very high precision measurements from multiple sites in separate hemispheres, may help constrain these parameters in future model studies. PC2 reflected the role of temperature increase, while PC3 was dominated the chosen fractionation factors, which drive loss partitioning in the soil submodule through the parameter frac_ex. Both the correlation and PCA analysis show that most of the optimized parameters - particularly those related to isotopic composition - can be sufficiently discriminated by the model-data assimilation. Parameters only constrained by mixing ratio cannot be distinguished easily, reflecting the importance of isotopic data for constraining the global N cycle.

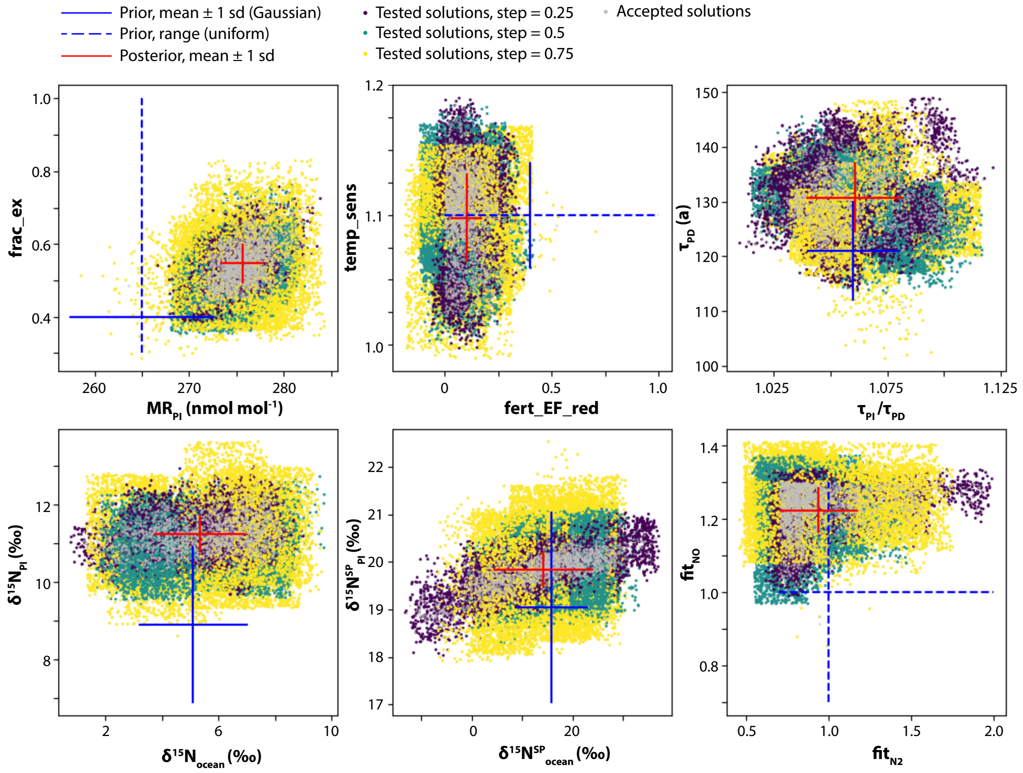

Supplementary Figure 7: Tested and accepted model solutions from the MCMC optimization of a coupled soil-atmosphere model of N_2O emissions and isotopic composition. Parameter descriptions and references for prior estimates are given in Supplementary Table 1.

The MCMC approach led to a strong improvement in model-observation agreement, with RMSE decreasing from 14.1 to 1.7 nmol mol^{-1} for N_2O mixing ratio, 1.5 to 0.5‰ for $\delta^{15}N$, 1.5 to 1.1‰ for $\delta^{15}N^{SP}$, and 5.4 to 1.8% for climate-zone emission factor (Supplementary Fig. 9). The improvement in $\delta^{15}N^{SP}$ is lower than for $\delta^{15}N$ because measurements are more uncertain relative to the trend, and the timeseries is much shorter. Prior observation EFs were highly uncertain, and therefore EFs were the only observation parameter showing a significant difference between the prior and the posterior. Posterior observation EFs were higher than prior observations EFs, which is likely due to the highly dynamic nature of N_2O emissions, whereby emission peaks

Supplementary Table 1: Prior and posterior values and uncertainty ranges for model input parameters optimized using the MCMC approach. All parameters are defined in the Methods. 'Abb.' is the abbreviation for the parameter used throughout this paper. 'Ref' is the citation for the prior estimate. 'PDF' is the probability density function: G = Gaussian, U = uniform. References: References: 1. [30], 2. [23], 3. [33], 4. [22], 5. [29], 6. [34], 7. [32], 8. [26].

Abb.	Description	Unit	Prior	Ref.	Posterior	PDF
MR_{PI}	N_2O mixing ratio for the preindustrial (PI) troposphere	nmol mol^{-1}	265±7.5	1,2	276±2.2	G
frac_ex	Fractionation expression factor	Unitless	0.4 (0.3-1.0)	NA	0.55±0.05	U
fert_EF_red	EF reduction for fertiliser emissions	Unitless	0.4 (0-1.0)	NA	0.30±0.07	U
temp_sens	Temperature sensitivity of N_2O emissions (as a fraction of preindustrial emissions)	f_{PI} °C^{-1}	1.1±0.04	3	1.10±0.03	G
τ_{PI}/τ_{PD}	Ratio of N_2O lifetime (τ) in the pre-industrial and the present day (PD)	Unitless	1.06±0.02	4	1.06±0.02	G
τ_{PD}	N_2O τ in the PD	years	116±9	4	131±6	G
$\delta^{15}N_{ocean}$	Mean $\delta^{15}N$ bulk for the ocean source	‰	5.1±1.9	5	5.3±1.6	G
$\delta^{15}N_{PI}$	Mean $\delta^{15}N$ bulk for the PI troposphere	‰	8.9±2.0	6	11.2±0.3	G
$\delta^{15}N^{SP}_{ocean}$	Mean $\delta^{15}N^{SP}$ for the ocean source	‰	15.8±7.1	5	14.2±9.6	G
$\delta^{15}N^{SP}_{PI}$	Mean $\delta^{15}N^{SP}$ for the PI troposphere	‰	19.1±2.0	6	19.8±0.3	G
fit_{N2}	Scaling factor for the parameterisation of N_2 as a fraction of total N gas emissions	Unitless	1 (0.7-2)	NA	0.9±0.2	U
fit_{NO}	Scaling factor for the parameterisation of NO as a fraction of total N gas emissions	Unitless	1 (0.7-1.3)	NA	1.2±0.1	U
F_{ocean}	Total marine N_2O flux	Tg N_2O-N a^{-1}	5.1±1.8	7	5.0±0.9	G
T_to_S	Exchange rate between the troposphere and stratosphere	$\times 10^{17}$ kg a^{-1}	5.4 (4.1-6.6)	8	4.1±1.1	U

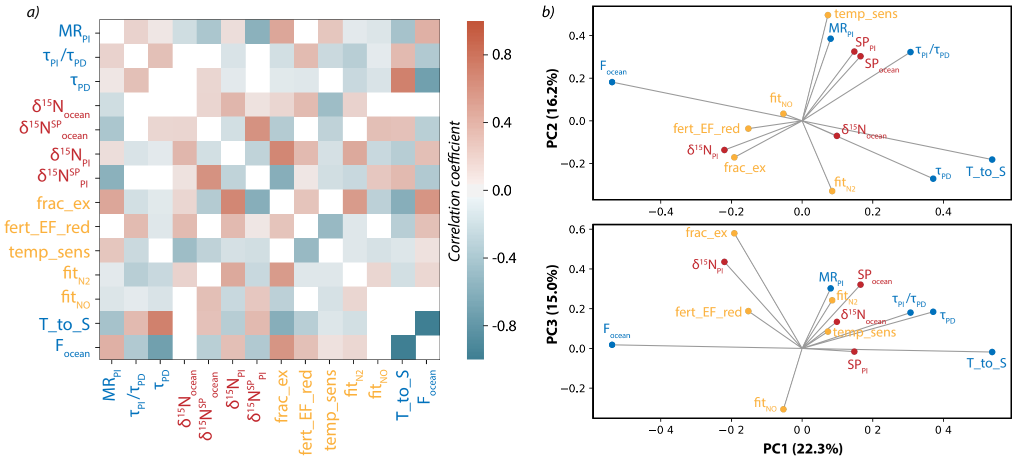

Supplementary Figure 8: Relationships between accepted solutions of parameters optimized with MCMC. *a*) Color map showing Pearson correlation coefficients between the specified parameters as indicated with the color bar. Only correlations significant at $p < 0.01$ are shown. *b*) Principal components analysis of all accepted solutions; soil and emissions module parameters are shown in orange, atmospheric parameters in blue, and isotopic parameters in red.

are not captured by most annual estimates which sample only weekly or biweekly [4, 36, 7]. Moreover, measured EFs are often based only on growing season emissions, which leads to a strong underestimation of emissions in cold regions [37, 5, 10]. Model and observation posterior EFs agreed well for most climate zones, although in the wetter climate zones agreement was worse, with modelled EFs were higher than observations. Emissions are highly variable and dynamic in the wetland and tropical regions [18, 24, 2], thus it is very challenging for observational studies to accurately capture annual emissions, which may account for this mismatch in these regions.

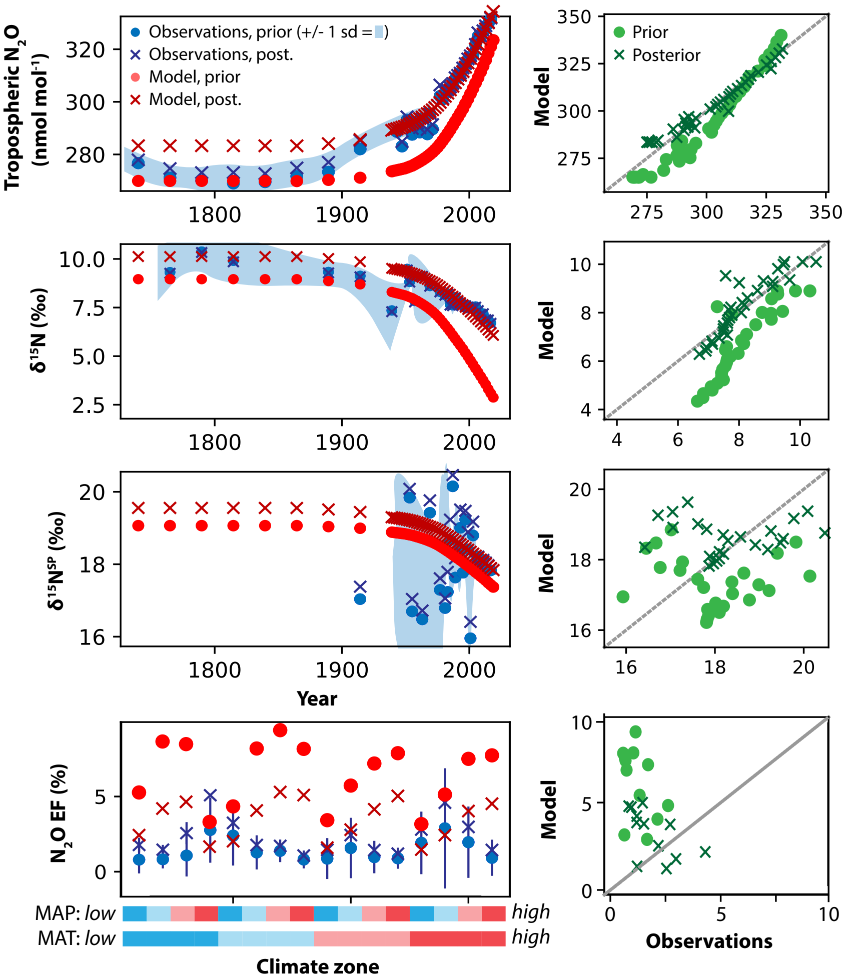

Supplementary Figure 9: Modelled N_2O mixing ratio, isotopic composition, and emission factors using prior and posterior estimates of the parameters shown in Supplementary Table 1. Left-hand panels shown model results and observations plotted against time or climate zone, and right-hand panels show a 1:1 comparison of model results and observations. The 1σ uncertainty in observations is shown as the blue shaded area for the upper three panels. The dotted grey line shows a 1:1 relationship. For EFs (bottom panel) both the prior and posterior estimates are shown with 1σ error bars, as the posterior estimates following the MCMC are significantly different to the prior estimates.

Supplementary Note 4: Partitioning N_2O emissions as nitrification and denitrification

Total N losses to nitrification were estimated as the sum of NO emissions and nitrification N_2O emissions per grid cell, and denitrification total N losses were similarly estimated as the sum of N_2 emissions and denitrification N_2O emissions [3] (Supplementary Fig. 10). Summing across all grid cells therefore allowed calculation of total nitrification and denitrification N losses over time, as well as estimation of the proportion of total N losses from each pathway, and the contribution of each pathway to N_2O emissions. The proportion of total N inputs lost to both pathways began decreasing around 1945, when fertilisation began to increase, as a significant amount of fertiliser N is removed and relocated via harvest. Since 1990, nitrification and denitrification loss fractions have slightly increased, as N inputs move from temperate to tropical regions which favour N gas production (Supplementary Fig. 10).

Total N losses to nitrification were somewhat lower than to denitrification, totalling 25.2±1.8 and 21.2±1.7 Tg-N a^{-1} in 2020 respectively. These are lower than suggested by a recent studies [27, 25], which estimated total terrestrial denitrification N losses of 90-135 Tg-N a^{-1}. Our estimate of the mean terrestrial denitrification product ratio ($R_{N2O} = \frac{N_2O_{denit}}{N_2O_{denit}+N_2}$) of 27±4% in 2020 (26±3% in 1850) agrees very well with the estimate of 23% based on a meta-analysis of experimental studies by [27]. Wetland and estuarine soils are not represented in the $\delta^{15}N$ database used as the basis for modelling in this study, suggesting these regions may be hotspots for complete denitrification and N_2 production, accounting for the discrepancy in modelled total denitrification. Total denitrification N losses are highly uncertain due to the technical challenge of making accurate annual measurements of N_2 fluxes, thus model validation is very difficult (e.g. Supplementary Fig. 6). An improved quantitative understanding of the drivers of denitrification, as well as measurements of soil $\delta^{15}N$ in wetland and estuarine environments, will be key to achieving robust model estimates of total global denitrification.

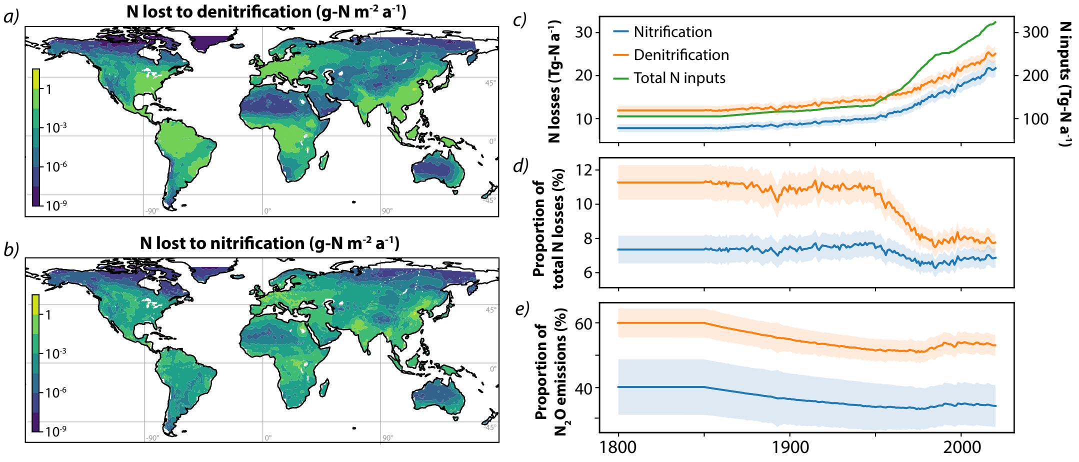

Supplementary Figure 10: N losses to nitrification and denitrification. *a*, *b*) Global map of N losses in 2020 on a $0.5 \times 0.5^\circ$ degree grid via denitrification (*a*) and nitrification (*b*). *c*) Total N losses to nitrification and denitrification per year from 1800 to 2020 (left axis) compared to total terrestrial N inputs (right axis). *d*) Proportion of total terrestrial N inputs lost to nitrification and denitrification from 1800 to 2020. *e*) Proportion of terrestrial N_2O emissions contributed by nitrification and denitrification from 1800 to 2020 (remaining N_2O emissions are from EDGAR categories 1A1, 1A3b, 2B and 6, see Methods). In all panels the shaded area indicates the 1σ uncertainty. Maps generated with Cartopy (Met Office, 2015, [20]).

Supplementary Note 5: Estimation of anthropogenic N_2O flux and isotopic composition

The anthropogenic N_2O flux was calculated by assuming that all emission increases since 1850 are anthropogenic (Supplementary Fig. 11). Most of the input datasets did not include data before 1850, so it was not possible to calculate a mean pre-anthropogenic baseline for either mixing ratio or isotopic composition over several decades prior to 1850, or to account for possible anthropogenic influences before 1850. Although natural emissions would show some variability due to natural climate fluctuations, natural emission variability is expected to be much lower than the key anthropogenic influences of climate change and agriculture [32]. This method of estimating the total anthropogenic N_2O flux includes both direct and indirect emissions, as well as increased emissions from natural ecosystems due to anthropogenic climate change feedbacks, and is thus not directly comparable with many other studies that report only the flux directly accounted for by anthropogenic N inputs.

The isotopic composition of the anthropogenic source was found using:

$$\delta_{\mathrm{total}} = f_{\mathrm{a}} \times \delta_{\mathrm{a}} + (1 - f_{\mathrm{a}}) \times \delta_{\mathrm{n}} \tag{7}$$

where δ represents either δ^{15}N or $\delta^{15}N^{SP}$ of N_2O, 'total' designates the total source isotopic composition (directly modelled as the sum of fluxes across all gridcells), subscripts 'a' and 'n' refer to anthropogenic and natural sources respectively, and f_{a} is the fraction of N_2O from anthropogenic sources. Anthropogenic source isotopic composition was found for years where anthropogenic emissions contribute at least 10% of the total flux.

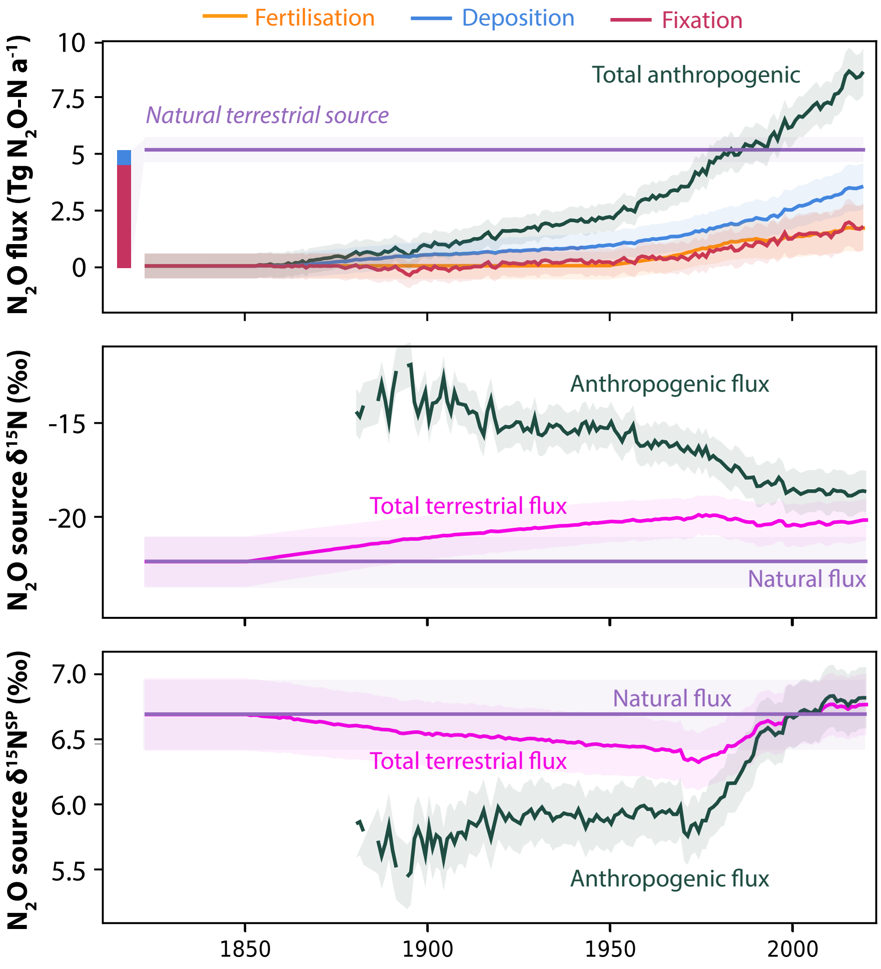

Supplementary Figure 11: Anthropogenic and natural terrestrial N_2O emissions and isotopic composition. The top panel shows the anthropogenic flux broken down into N input categories of fertilisation, deposition and fixation, estimated by assuming that all increases in N_2O emissions for all input categories (fixation, fertilisation, deposition) after 1850 are due to anthropogenic influences. Total N_2O emissions, including non-soil N_2O emissions, are also shown (blue line). The bars at the left indicate the breakdown of natural emissions driven by deposition and fixation. The bottom panels show the $\delta^{15}N$ and $\delta^{15}N^{SP}$ for natural and anthropogenic emissions and for total terrestrial emissions. N inputs with time as well as growth rate of N_2O emissions for each input category are shown in Figure 2 of the main article. The shaded areas indicate the 1σ uncertainty.

Supplementary figures 12-14

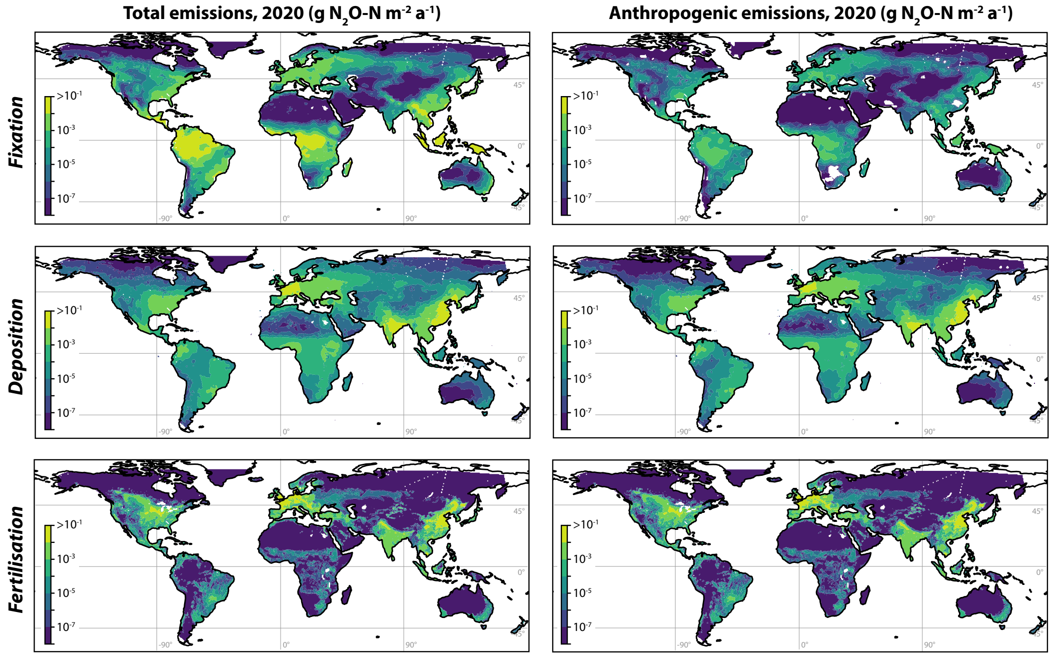

Supplementary Figure 12: Modelled terrestrial (natural + anthropogenic) N_2O soil emissions from different input types. The left panels show total emissions for inputs from biological N fixation, N deposition, and N fertilisation for the year 2020; all three subpanels have the same colour scale. The right panels show anthropogenic emissions for each input type in 2020, estimated by subtracting total emissions for the year 1850 from total 2020 emissions. All subpanels have the same colour scale. Maps generated with Cartopy (Met Office, 2015, [20]).

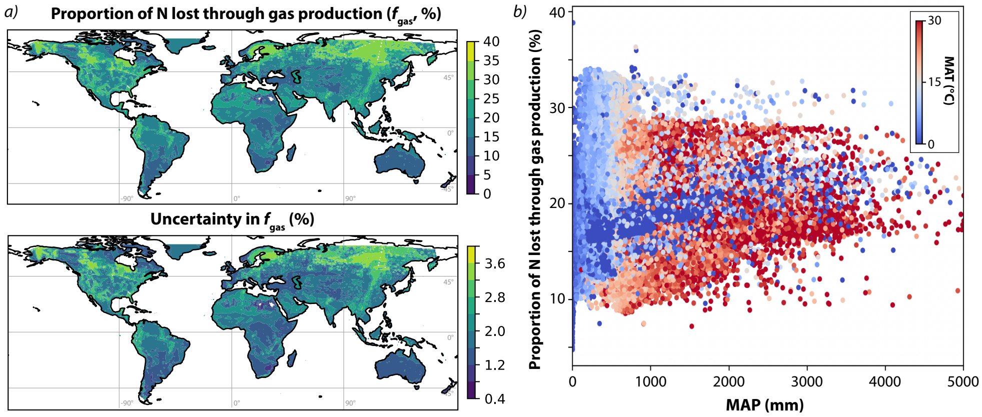

Supplementary Figure 13: Modelled proportion of N lost through gas production: *a*) Global gridded f_{gas} (upper) and the 1σ uncertainty (lower); *b*) Relationship between mean annual precipitation, mean annual temperature (point colour) and f_{gas} for each grid cell. Maps generated with Cartopy (Met Office, 2015, [20]).

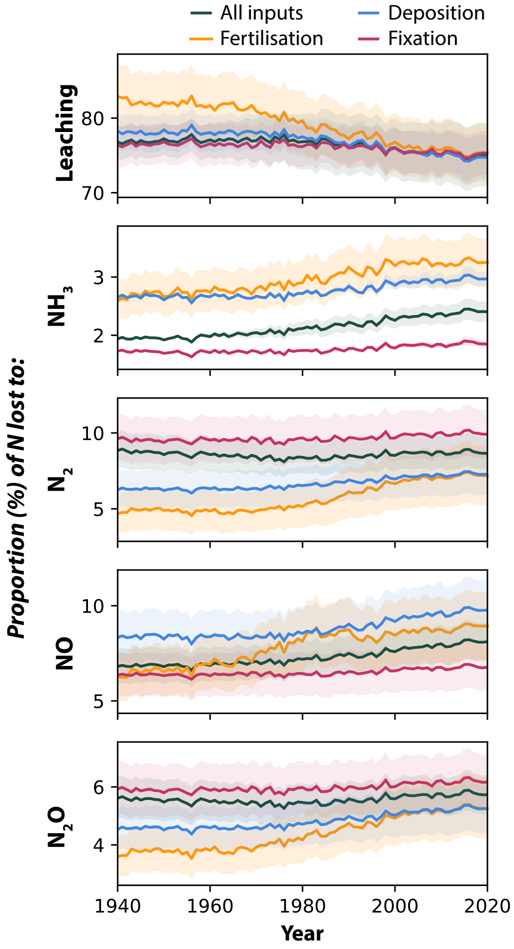

Supplementary Figure 14: The simulated proportion of N from different input categories (fertilisation, deposition, fixation) that is lost via leaching, NH_3 volatilization, and gas production (EFs for N_2, N_2O, NO) in soils from 1940-2020.

References

[1] R. Amundson, A. T. Austin, E. A. Schuur, K. Yoo, V. Matzek, C. Kendall, A. Uebersax, D. Brenner, and W. T. Baisden. Global patterns of the isotopic composition of soil and plant nitrogen. *Global Biogeochemical Cycles*, 17(1), 2003.

[2] C. Arias-Navarro, E. Díaz-Pinés, S. Klatt, P. Brandt, M. C. Rufino, K. Butterbach-Bahl, and L. V. Verchot. Spatial variability of soil N2O and CO2 fluxes in different topographic positions in a tropical montane forest in Kenya. *Journal of Geophysical Research: Biogeosciences*, 122(3):514–527, 2017.

[3] E. Bai, B. Z. Houlton, and Y. P. Wang. Isotopic identification of nitrogen hotspots across natural terrestrial ecosystems. *Biogeosciences*, 9(8):3287–3304, 2012.

[4] K. Butterbach-Bahl, E. M. Baggs, M. Dannenmann, R. Kiese, and S. Zechmeister-Boltenstern. Nitrous oxide emissions from soils: how well do we understand the processes and their controls? *Philosophical transactions of the Royal Society of London, Series B, Biological sciences*, 368(1621), 2013.

[5] E. Byers, M. A. Bleken, and P. Dörsch. Winter N2O accumulation and emission in sub-boreal grassland soil depend on clover proportion and soil ph. *Environmental Research Communications*, 3(1), 2021.

[6] F. Chollet. Keras, 2015.

[7] K. A. Congreves, C. Wagner-Riddle, B. C. Si, and T. J. Clough. Nitrous oxide emissions and biogeochemical responses to soil freezing-thawing and drying-wetting. *Soil Biology and Biochemistry*, 117(October 2017):5–15, 2018.

[8] J. M. Craine, A. J. Elmore, L. Wang, L. Augusto, W. T. Baisden, E. N. Brookshire, M. D. Cramer, N. J. Hasselquist, E. A. Hobbie, A. Kahmen, K. Koba, J. M. Kranabetter, M. C. Mack, E. Marin-Spiotta, J. R. Mayor, K. K. McLauchlan, A. Michelsen, G. B. Nardoto, R. S. Oliveira, S. S. Perakis, P. L. Peri, C. A. Quesada, A. Richter, L. A. Schipper, B. A. Stevenson, B. L. Turner, R. A. Viani, W. Wanek, and B. Zeller. Convergence of soil nitrogen isotopes across global climate gradients. *Scientific Reports*, 5:1–8, 2015.

[9] X. Cui, F. Zhou, P. Ciais, E. A. Davidson, F. N. Tubiello, X. Niu, X. Ju, J. G. Canadell, A. F. Bouwman, R. Jackson, N. Mueller, X. Zheng, D. Kanter, H. Tian, W. Adalibieke, Y. Bo, Q. Wang, X. Zhan, and D. Zhu. Global mapping of crop-specific emission factors highlights hotspots of nitrous oxide mitigation. *Nature Food*, In press, 2021.

[10] P. Doersch, I. Sturite, and S. Trier Kjaer. High off-season nitrous oxide emissions negate potential soil C-gain from cover crops in boreal cereal cropping (EGU22-3066). *EGU General Assembly 2022*, 2022.

[11] C. Dorich, R. Conant, F. Albanito, K. Butterbach-Bahl, P. Grace, C. Scheer, V. Snow, I. Vogeler, and T. van der Weerden. Improving N2O emission estimates with the global N2O database. *Current Opinion in Environmental Sustainability*, 47:13–20, 2020.

[12] E. Harris, E. Diaz-Pines, E. Stoll, M. Schloter, S. Schulz, C. Duffner, K. Li, K. Moore, J. Ingrisch, D. Reinthaler, S. Zechmeister-Boltenstern, S. Glatzel, and M. Bahn. Denitrifying pathways dominate nitrous oxide emissions from managed grassland during drought and rewetting. *Science Advances*, 7(6):eabb7118, 2021.

[13] E. Harris, T. Ladreiter-Knauss, K. Butterbach-Bahl, B. Wolf, and M. Bahn. Land-use and abandonment alters methane and nitrous oxide fluxes in mountain grasslands. *Science of the Total Environment*, 628-629:997–1008, 2018.

[14] L. Hoertnagl, M. Barthel, N. Buchmann, W. Eugster, K. Butterbach-bahl, E. Diaz-Pines, M. Zeeman, K. Klumpp, R. Kiese, M. Bahn, A. Hammerle, H. Lu, T. Ladreiter-Knauss, S. Burri, and L. Merbold. Greenhouse gas fluxes over managed grasslands in Central Europe. *Global Change Biology*, 24:1843–1872, 2018.

[15] H. W. Hu, D. Chen, and J. Z. He. Microbial regulation of terrestrial nitrous oxide formation: Understanding the biological pathways for prediction of emission rates. *FEMS Microbiology Reviews*, 39(5):729–749, 2015.

[16] E. Ibraim, B. Wolf, E. Harris, R. Gasche, J. Wei, L. Yu, R. Kiese, S. Eggleston, K. Butterbach-Bahl, M. Zeeman, B. Tuzson, L. Emmenegger, J. Six, S. Henne, and J. Mohn. Attribution of N2O sources in a grassland soil with laser spectroscopy based isotopocule analysis. *Biogeosciences*, 16:3247–3266, 2019.

[17] M. Inatomi, T. Hajima, and A. Ito. Fraction of nitrous oxide production in nitrification and its effect on total soil emission: A meta-analysis and global-scale sensitivity analysis using a process-based model. *Plos One*, 14(7):e0219159, 2019.

[18] E. A. Kort, P. K. Patra, K. Ishijima, B. C. Daube, R. Jiménez, J. Elkins, D. Hurst, F. L. Moore, C. Sweeney, and S. C. Wofsy. Tropospheric distribution and variability of N2O: Evidence for strong tropical emissions. *Geophysical Research Letters*, 38:1–5, 2011.

[19] Z. Luo, R. A. Viscarra-Rossel, and T. Qian. Similar importance of edaphic and climatic factors for controlling soil organic carbon stocks of the world. *Biogeosciences*, 18(6):2063–2073, 2021.

[20] Met Office. Cartopy: a cartographic python library with a Matplotlib interface (https://scitools.org.uk/cartopy), 2015.

[21] K. Pilegaard. Processes regulating nitric oxide emissions from soils. *Philosophical Transactions of the Royal Society B: Biological Sciences*, 368(1621), 2013.

[22] M. J. Prather, J. Hsu, N. M. Deluca, C. H. Jackman, L. D. Oman, A. R. Douglass, E. L. Fleming, S. E. Strahan, S. D. Steenrod, O. A. Søvde, I. S. a. Isaksen, L. Froidevaux, and B. Funke. Measuring and modeling the lifetime of nitrous oxide including its variability. *Journal of Geophysical Research - Atmospheres*, 120:5693–5705, 2015.

[23] T. Röckmann, J. Kaiser, and C. A. M. Brenninkmeijer. The isotopic fingerprint of the pre-industrial and the anthropogenic N2O source. *Atmospheric Chemistry and Physics*, 3:315–323, 2003.

[24] D. W. Rowlings, P. R. Grace, C. Scheer, and S. Liu. Rainfall variability drives interannual variation in N2O emissions from a humid, subtropical pasture. *Science of the Total Environment*, 512-513:8–18, 2015.

[25] C. Scheer, K. Fuchs, D. E. Pelster, and K. Butterbach-Bahl. Estimating global terrestrial denitrification from measured N2O:(N2O + N2) product ratios. *Current Opinion in Environmental Sustainability*, 47:72–80, 2020.

[26] A. Schilt, E. J. Brook, T. K. Bauska, D. Baggenstos, H. Fischer, F. Joos, V. V. Petrenko, H. Schaefer, J. Schmitt, J. P. Severinghaus, R. Spahni, and T. F. Stocker. Isotopic constraints on marine and terrestrial N2O emissions during the last deglaciation. *Nature*, 516(7530):234–237, 2014.

[27] W. H. Schlesinger. On the fate of anthropogenic nitrogen. *Proceedings of the National Academy of Sciences of the United States of America*, 106(1):203–208, 2009.

[28] D. Sihi, E. A. Davidson, K. E. Savage, and D. Liang. Simultaneous numerical representation of soil microsite production and consumption of carbon dioxide, methane, and nitrous oxide using probability distribution functions. *Global Change Biology*, 26(1):200–218, 2020.

[29] D. M. Snider, J. J. Venkiteswaran, S. L. Schiff, and J. Spoelstra. From the Ground Up: Global Nitrous Oxide Sources are Constrained by Stable Isotope Values. *Plos One*, 10(3), 2015.

[30] T. Sowers, A. Rodebaugh, N. Yoshida, and S. Toyoda. Extending records of the isotopic composition of atmospheric N2O back to 1800 A.D. from air trapped in snow at the South Pole and the Greenland Ice Sheet Project II ice core. *Global Biogeochem. Cycles*, 16(4):1129, 2002.

[31] R. L. Thompson, L. Lassaletta, P. K. Patra, C. Wilson, K. C. Wells, A. Gressent, E. N. Koffi, M. P. Chipperfield, W. Winiwarter, E. A. Davidson, H. Tian, and J. Canadell. Acceleration of global N2O emissions seen from two decades of atmospheric inversion. *Nature Climate Change*, page 8, 2019.

[32] H. Tian, R. Xu, J. G. Canadell, R. L. Thompson, W. Winiwarter, P. Suntharalingam, E. A. Davidson, P. Ciais, R. B. Jackson, G. Janssens-maenhout, M. J. Prather, P. Regnier, N. Pan, S. Pan, G. P. Peters, H. Shi, F. N. Tubiello, S. Zaehle, F. Zhou, A. Arneth, G. Battaglia, S. Berthet, L. Bopp, A. F. Bouwman, E. T. Buitenhuis, J. Chang, M. P. Chipperfield, S. R. S. Dangal, E. Dlugokencky, J. W. Elkins, B. D. Eyre, B. Fu, B. Hall, A. Ito, F. Joos, P. B. Krummel, A. Landolfi, G. G. Laruelle, R. Lauerwald, W. Li, S. Lienert, T. Maavara, M. Macleod, D. B. Millet, S. Olin, P. K. Patra, R. G. Prinn, P. A. Raymond, D. J. Ruiz, G. R. Werf, N. Vuichard, J. Wang, R. F. Weiss, K. C. Wells, C. Wilson, J. Yang, and Y. Yao. A comprehensive quantification of global nitrous oxide sources and sinks. *Nature*, 586, 2020.

[33] H. Tian, J. Yang, R. Xu, C. Lu, J. G. Canadell, E. A. Davidson, R. B. Jackson, A. Arneth, J. Chang, P. Ciais, S. Gerber, A. Ito, F. Joos, S. Lienert, P. Messina, S. Olin, S. Pan, C. Peng, E. Saikawa, R. L. Thompson, N. Vuichard, W. Winiwarter, S. Zaehle, and B. Zhang. Global soil nitrous oxide emissions since the preindustrial era estimated by an ensemble of terrestrial biosphere models: Magnitude, attribution, and uncertainty. *Global Change Biology*, 25(2):640–659, 2019.

[34] S. Toyoda, N. Kuroki, N. Yoshida, K. Ishijima, Y. Tohjima, and T. Machida. Decadal time series of tropospheric abundance of N2O isotopomers and isotopologues in the northern hemisphere obtained by the long-term observation at Hateruma Island, Japan. *Journal of Geophysical Research - Atmospheres*, 118:1–13, 2013.

[35] E. Verhoeven, M. Barthel, L. Yu, L. Celi, D. Said-Pullicino, S. Sleutel, D. Lewicka-Szczebak, J. Six, and C. Decock. Early season N2O emissions under variable water management in rice systems: Source-partitioning emissions using isotope ratios along a depth profile. *Biogeosciences*, 16(2):383–408, 2019.

[36] C. Wagner-Riddle, E. M. Baggs, T. J. Clough, K. Fuchs, and S. O. Petersen. Mitigation of nitrous oxide emissions in the context of nitrogen loss reduction from agroecosystems: managing hot spots and hot moments. *Current Opinion in Environmental Sustainability*, 47:46–53, 2020.

[37] C. Wagner-Riddle, K. A. Congreves, D. Abalos, A. A. Berg, S. E. Brown, J. T. Ambadan, X. Gao, and M. Tenuta. Globally important nitrous oxide emissions from croplands induced by freeze-thaw cycles. *Nature Geoscience*, 10(4):279–283, 2017.

[38] B. Wolf, L. Merbold, C. Decock, B. Tuzson, E. Harris, J. Six, L. Emmenegger, and J. Mohn. First on-line isotopic characterization of N2O emitted from intensively managed grassland. *Biogeosciences*, 12:2517–2531, 2015.

[39] B. Wolf, X. Zheng, N. Brüggemann, W. Chen, M. Dannenmann, X. Han, M. a. Sutton, H. Wu, Z. Yao, and K. Butterbach-Bahl. Grazing-induced reduction of natural nitrous oxide release from continental steppe. *Nature*, 464(7290):881–4, apr 2010.

[40] L. Xia, X. Li, Q. Ma, S. K. Lam, B. Wolf, R. Kiese, K. Butterbach-Bahl, D. Chen, Z. Li, and X. Yan. Simultaneous quantification of N2, NH3 and N2O emissions from a flooded paddy field under different N fertilization regimes. *Global Change Biology*, (December 2019):1–12, 2019.

[41] L. Yu, E. Harris, D. Lewicka-Szczebak, and J. Mohn. What can we learn from N2O isotope data? Analytics, processes and modelling. *Rapid Communications in Mass Spectrometry*, 2020.
